# A unique Z-shaped tetramer mediates the autoinhibition of waterfowl STING

**DOI:** 10.1371/journal.ppat.1014111

**Published:** 2026-04-08

**Authors:** Zhenchao Zhao, Xiangyu Huang, Lei Wu, Xin Li

**Affiliations:** State Key Laboratory of Veterinary Public Health and Safety, College of Veterinary Medicine, China Agricultural University, Beijing, China; Florida State University, UNITED STATES OF AMERICA

## Abstract

Stimulator of interferon genes (STING) is a central player of innate immunity, coordinating host defense against viral infection and cancer. While the canonical architectures of apo-STING and ligand-bound STING have been established, current knowledge is limited to a subset of species, and a comprehensive cross-species, ligand-resolved structural atlas remains incomplete. Here, we determined the high-resolution crystal structures of duck and bovine STING ligand-binding domains (LBDs) bound to 2′3′-cGAMP and of duck, bovine, and human STING LBDs bound to the non-nucleotide agonist diABZI3. In the 2′3′-cGAMP complexes, the lid regions were ordered and completely covered the ligand-binding pocket, whereas in the diABZI3 complexes, the lid regions were completely disordered. In human and bovine STING, 2′3′-cGAMP induced a more closed dimer conformation than diABZI3, while duck STING exhibited minimal differences in closure between the two ligands. Strikingly, non-reducing SDS-PAGE revealed a distinct disulfide-linked tetramer in duck STING, which is abolished by the C195S mutation. Within the crystal lattice of the duck STING LBD–2′3′-cGAMP complex, we observed a unique Z-shaped tetramer stabilized by an interfacial disulfide bond between C195 and a network of polar interactions. Disrupting this interface, either by the C195S mutation or by ligand stimulation with 2′3′-cGAMP or diABZI3, relieved the tetrameric constraint and amplified STING signaling, establishing this tetramer as a duck-specific autoinhibitory assembly. These findings expand the structural repertoire of STING oligomeric assemblies, fill the structural gap for duck STING, and provide a comparative structural framework for species-specific STING regulation.

## Author summary

STING signaling is governed by its oligomeric assembly, yet the nature and mechanisms of species-specific assemblies remain unclear. Here we present high-resolution crystal structures of duck, bovine STING LBDs bound to 2′3′-cGAMP, and duck, bovine, and human STING LBDs bound to diABZI3, elucidating how these ligands induce conformational changes and lid ordering in mammals versus waterfowl. Strikingly, we identify a previously unrecognized Z-shaped tetramer in duck STING, bridged by a C195-C195 disulfide bond, which is also present in waterfowl but absent in chicken and mammals. This quaternary structure differs from the known autoinhibited head-to-head bilayer assembly and activated side-by-side polymeric asseblies, and likely reflects a unique autoinhibitory mechanism in waterfowl STING. Overall, our findings broaden the known structural repertoire of STING oligomeric assemblies and establish a comparative framework for understanding species-specific regulation of STING activation.

## Introduction

Stimulator of interferon genes (STING, also known as TMEM173 [1], MITA [2], MPYS [3], or ERIS [4]) is an adaptor of the cytosolic DNA-sensing pathway mediated by cyclic GMP-AMP synthase (cGAS). The cGAS-STING pathway is critical for innate immune defense against infection and cancer [5,6]. Upon detecting pathogenic or damaged DNA in the cytosol, cGAS catalyzes the synthesis of the second messenger, 2′3′-cGAMP, which binds and activates endoplasmic reticulum (ER)-resident STING [7,8]. Ligand engagement drives ER-to-Golgi trafficking, promotes high-order oligomerization of STING, and recruits TANK-binding kinase 1 (TBK1) and interferon regulatory factor 3 (IRF3), as well as engaging NF-κB signaling, ultimately inducing type I interferon (IFN-I) and multiple pro-inflammatory cytokines to strengthen antimicrobial and antitumor immunity [9–13].

Dysregulated or chronic STING activation provokes pathological inflammation and autoimmunity [14]. Monogenic autoinflammatory disorders such as Aicardi-Goutieres syndrome (AGS) [15] and STING-associated vasculopathy with onset in infancy (SAVI) [16,17] are tightly linked to hyperactive STING signaling. Conversely, in cancer immunotherapy, STING activation synergizes with immune checkpoint blockade to promote durable antitumor responses [18,19]. These dual roles highlight the need for structural insights into STING activation, autoinhibition, and oligomeric assembly.

STING comprises an N-terminal transmembrane bundle, a cytosolic ligand-binding domain (LBD), and a flexible C-terminal tail (CTT) that recruits and activates TBK1/IRF3 [20–22]. Structural studies show that 2′3′-cGAMP binding induces inward rigid-body rotation of the LBD protomers, shrinking the dimer pocket and driving a transition from an “open” to a “closed” conformation [23]. This closed state couples to about 180° LBD rotation relative to the transmembrane domain (TMD) in the full-length STING structure by cryo-electron microscopy (cryo-EM) [20]. This conformational change promotes interactions between STING dimers and drives their assembly into linear oligomers [20,24]. Consequently, the arrays provide a structural platform for TBK1 recruitment and trans-phosphorylation of CTT between neighboring dimers [21,25]. In the absence of ligand, STING adopts self-inhibited head-to-head arrangements that occlude downstream factor binding [24]. Consistent with crystal packing, 2′3′-cGAMP bound STING LBD complexes can extend laterally into high-order oligomers within the crystal lattice [26]. Notably, the high-potency exogenous agonist diABZI3 further diversifies oligomeric contacts, producing mixed head-to-head and side-by-side assemblies and heterogeneous activation kinetics [27].

To extend structural understanding of STING across species, we selected duck STING as an avian representative and compared it to bovine and human STING. Using the endogenous ligand 2′3′-cGAMP and the non-nucleotide agonist diABZI3, we determined high-resolution crystal structures of duck and bovine STING bound to 2′3′-cGAMP and of duck, bovine, and human STING bound to diABZI3. Biophysical and cellular assays corroborated the structural findings, revealing pronounced interspecies differences in dimer closure and oligomerization propensity. Importantly, duck STING adopts a unique Z-shaped tetrameric assembly stabilized by a C195-C195 disulfide and cooperative polar contacts. Disrupting this interface relieves autoinhibition and potentiates STING signaling. This finding broadens the known spectrum of STING oligomeric assemblies and highlights species-specific modes of STING regulation.

## Results

### Crystal structures of duck and bovine STING LBD bound to 2′3′-cGAMP

**Fig 1:**
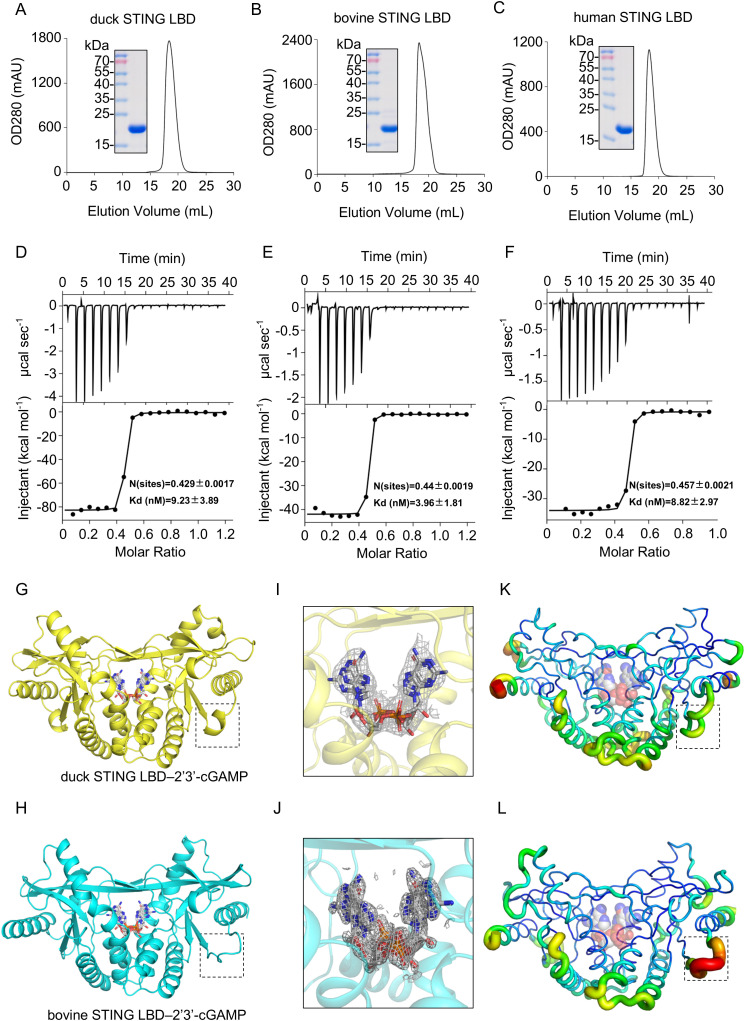
Crystal structures of 2′3′-cGAMP bound to duck and bovine STING ligand-binding domains (LBDs). **(A–C)** Gel-filtration chromatography of purified STING LBDs from duck **(A)**, bovine **(B)**, and human **(C)**. **(D–F)** Binding studies of 2′3′-cGAMP with duck STING LBD **(D)**, bovine STING LBD **(E)**, human STING LBD (**F**) by isothermal titration calorimetry (ITC). **(G)** Crystal structure of duck STING LBD in complex with 2′3′-cGAMP at 2.49 Å resolution. The symmetric STING dimer was shown as yellow cartoon representation, and 2′3′-cGAMP was shown as stick representation, bound within the inter-protomer cleft at the dimer interface. The black dashed box highlighted the region of residues 318–324. **(H)** Crystal structure of bovine STING LBD in complex with 2′3′-cGAMP at 1.60 Å resolution. The symmetric STING dimer was shown as a cyan cartoon representation, and 2′3′-cGAMP was shown as stick representation, bound within the inter-protomer cleft at the dimer interface. The black dashed box highlighted the region of residues 315–321. **(I)** The 2Fo–Fc electron-density map for 2′3′-cGAMP in the duck STING LBD–2′3′-cGAMP complex, contoured at 1.0σ post-refinement. Two alternative conformations of 2′3′-cGAMP were modeled at 0.5 occupancy each. **(J)** The 2Fo–Fc electron-density map for 2′3′-cGAMP in the bovine STING LBD–2′3′-cGAMP complex, contoured at 1.0σ post-refinement. Two alternative conformations of 2′3′-cGAMP were modeled at 0.5 occupancy each. **(K)** B-factor–colored representation of 2′3′-cGAMP -bound duck STING LBD, with high B-factors in red (thick ribbons) and low B-factors in green (thin ribbons). The black dashed box highlighted the region of residues 318–324. The ligand 2′3′-cGAMP was displayed as spheres representation. **(L)** B-factor–colored representation of 2′3′-cGAMP–bound bovine STING LBD, with high B-factors in red (thick ribbons) and low B-factors in green (thin ribbons). The black dashed box highlighted the region of residues 315–321. The ligand 2′3′-cGAMP was displayed as spheres representation.

Most structural studies of STING had primarily focused on mammals, particularly human STING [28–31]. In contrast, the high-resolution structure of avian STING, especially from *Anas platyrhynchos* (duck), has remained uncharacterized. Avian STING diverged substantially in both sequence and evolutionary lineage from mammalian counterparts and mediated distinct, species-specific innate immune responses [32] (S1A **and** S1B Fig **and** S1 Table). To probe the structural diversity and evolutionary conservation of STING, we also included *Bos taurus* (bovine) STING as a representative non-primate mammalian ortholog. We cloned, expressed, and purified the C-terminal ligand binding domains (LBDs) of duck, bovine and human STING in *E.*coli, and successfully obtained highly homogeneous recombinant proteins suitable for structural and functional analyses (**Fig 1A-1C**).

Subsequently, we measured the binding affinities of the LBDs of duck, bovine, and human STING for the endogenous cyclic dinucleotide agonist 2′3′-cGAMP by isothermal titration calorimetry (ITC). All three LBDs exhibited nanomolar binding affinities with dissociation constants (K_D_) of 9.2 nM for duck STING, 3.9 nM for bovine STING, and 8.8 nM for human STING, consistent with previous reports [8,23] (**Fig 1D-1F**). These results demonstrate that, despite considerable sequence and structural divergence, high-affinity recognition of endogenous ligand 2′3′-cGAMP is conserved across vertebrate STING, underscoring its central evolutionary role in innate immune signaling.

We co-crystallized duck and bovine STING LBDs with 2′3′-cGAMP, obtaining crystals in space groups P6₅22 (duck) and P2₁2₁2₁ (bovine). Structures were solved by molecular replacement using structural models generated by SWISS-MODEL and refined to 2.49 Å (duck) and 1.60 Å (bovine) resolution (S2 Table). In both crystals, the asymmetric unit contained one STING protomer, and application of crystallographic two-fold symmetry generated the canonical butterfly-like dimer (**Fig 1G and 1H**). The 2′3′-cGAMP ligand was positioned on the twofold axis. Because this axis intersects an intrinsically asymmetric ligand, 2′3′-cGAMP was modeled in two symmetry-related orientations, each refined at 0.5 occupancy (**Fig 1I and 1J**).

A notable difference emerged near the C terminus of the duck LBD: residues 318–324 (EELVEAE) formed a short α-helix, whereas the corresponding segments in bovine and human STING (residues 315–321, QEPAEGS/QEPADDS) adopted predominantly coil conformations (**Figs 1G**, **1H and** S1C). This difference was further supported by refined B-factor profiles, with the duck segment displaying lower and more uniform values (**Figs 1K**, **1L and** S1D). Sequence analysis explains this divergence: the duck STING segment is enriched in helix-promoting residues (E, L, A, V), whereas the bovine/human segments contain helix-disfavoring residues (P, D, G).

### Crystal structures of duck, bovine, human STING LBD in complex with diABZI3

**Fig 2:**
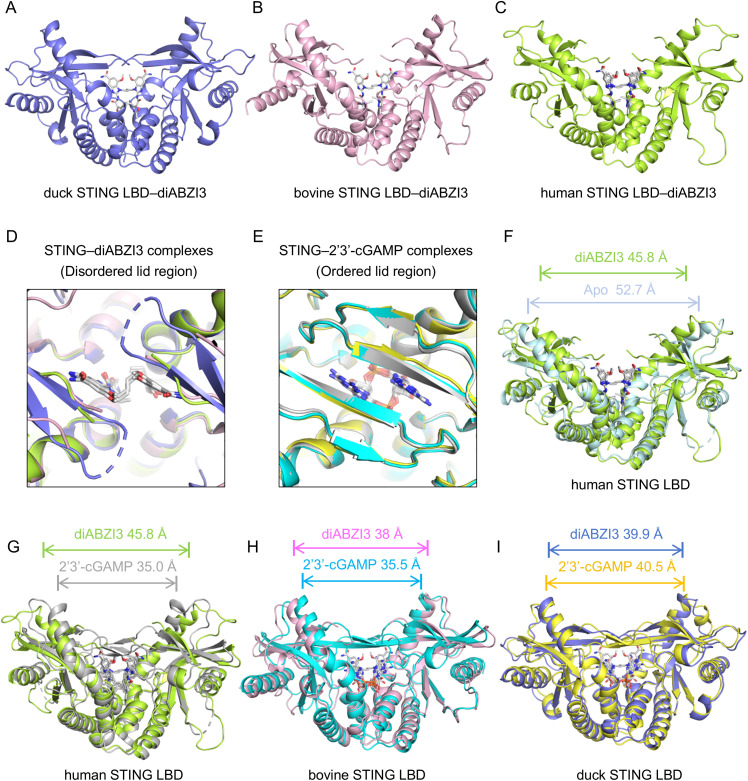
Comparison of 2′3′-cGAMP and diABZI3 bound to STING LBD complexes. **(A)** The crystal structure of diABZI3 bound to duck STING LBD at 1.65 Å. **(B)** The crystal structure of diABZI3 bound to bovine STING LBD at 1.81 Å. **(C)** The crystal structure of diABZI3 bound to human STING LBD at 1.9 Å. The symmetric STING dimers were shown as cartoons—duck in slate **(A)**, bovine in light pink **(B)**, and human in limon **(C)**,with diABZI3 rendered as sticks. **(D)** The lid region of diABZI3–bound STING LBD complexes from duck, bovine, and human. **(E)** The lid region of 2′3′-cGAMP–bound STING LBD complexes from duck, bovine, and human (PDB ID: 4KSY). **(F)** Superposition of diABZI3 bound to human STING LBD with both protomers in limon and apo-human STING LBD with both protomers in pale cyan (PDB ID: 6NT5). **(G)** Superposition of 2′3′-cGAMP bound to human STING LBD with both protomers in gray (PDB ID: 4KSY) and diABZI3 bound to human STING LBD with both protomers in limon. **(H)** Superposition of 2′3′-cGAMP bound to bovine STING LBD with both protomers in cyan and diABZI3–bound to bovine STING LBD with both protomers in light pink. **(I)** Superposition of 2′3′-cGAMP bound to duck STING LBD with both protomers in yellow and diABZI3 bound to duck STING LBD with both protomers in slate.

Since its discovery in 2019, diABZI3 has been recognized as a potent STING agonist, yet high-resolution structures of STING–diABZI3 complexes across species have remained scarce [33] (S2A Fig). To broaden structural coverage across species, we co-crystallized the LBDs of duck, bovine, and human STING with diABZI3, yielding crystals in space groups P4₁2₁2 (duck), P2₁2₁2₁ (bovine), and P6₁22 (human), respectively. Structures were solved by molecular replacement and refined to resolutions of 1.65 Å (duck), 1.81 Å (bovine), and 1.90 Å (human) (S2 Table). In all three complexes, diABZI3 occupied the hydrophobic cleft at the STING homodimer interface (**Fig 2A-2C**). The morpholine ring was highly flexible and poorly ordered in electron-density maps, precluding reliable modeling (S2B**-**S2D Fig).

A consistent feature of the diABZI3–bound STING LBD structures was the lack of interpretable density for the lid region (residues 233–240, duck numbering), which was therefore not included in the structural models, indicating conformational flexibility (**Fig 2D**). This observation is consistent with prior reports of STING complexes with diABZI analogs [27,33–35]. By contrast, in our 2′3′-cGAMP complexes (duck and bovine), the lid region was well ordered, with β-strands from both protomers pairing to form a four-stranded antiparallel β-sheet that covers the ligand-binding pocket and stabilizes the closed conformation (**Fig 2E**).

To assess the conformational changes in STING induced by 2′3′-cGAMP and diABZI3, we quantified the degree of closure by measuring the distance between the Cα atoms at the tips of the α1-helices from each monomer within the STING dimer. The reference residues were His185 in human STING, Phe186 in bovine STING, and Ala188 in duck STING [26] (S1B Fig). Superposition of the diABZI3-bound human STING complex with the apo-STING open conformation revealed a marked conformational difference (RMSD 2.42 Å). The tips of α1-helices separation decreased from approximately 52.7 Å in the open conformation to 45.8 Å in the diABZI3-bound STING complex, consistent with a moderate closure conformation reported previously [27] (**Fig 2F**). Comparison of 2′3′-cGAMP- and diABZI3-bound human STING structures showed an additional conformational difference (RMSD 1.77 Å), with 2′3′-cGAMP inducing a more closed conformation (35.0 Å) than diABZI3 (**Fig 2G**). A similar pattern was observed in bovine STING, where 2′3′-cGAMP- and diABZI3-bound bovine STING complexes differed by an RMSD of 1.29 Å. The α1-helices tips separation measured approximately 35.5 Å in the 2′3′-cGAMP complex versus 38.0 Å in the diABZI3 complex, indicating that 2′3′-cGAMP promotes a more closed conformation (**Fig 2H**). By contrast, duck STING displayed minimal differences between the two ligand-bound states: superposition of the complexes yielded an RMSD of 0.96 Å, with α1-helices tips separations of approximately 40.5 Å (2′3′-cGAMP) and 39.9 Å (diABZI3), suggesting that ligand binding induces only minor conformational changes in duck STING (**Fig 2I**).

### Structural basis of ligand recognition by duck STING

**Fig 3:**
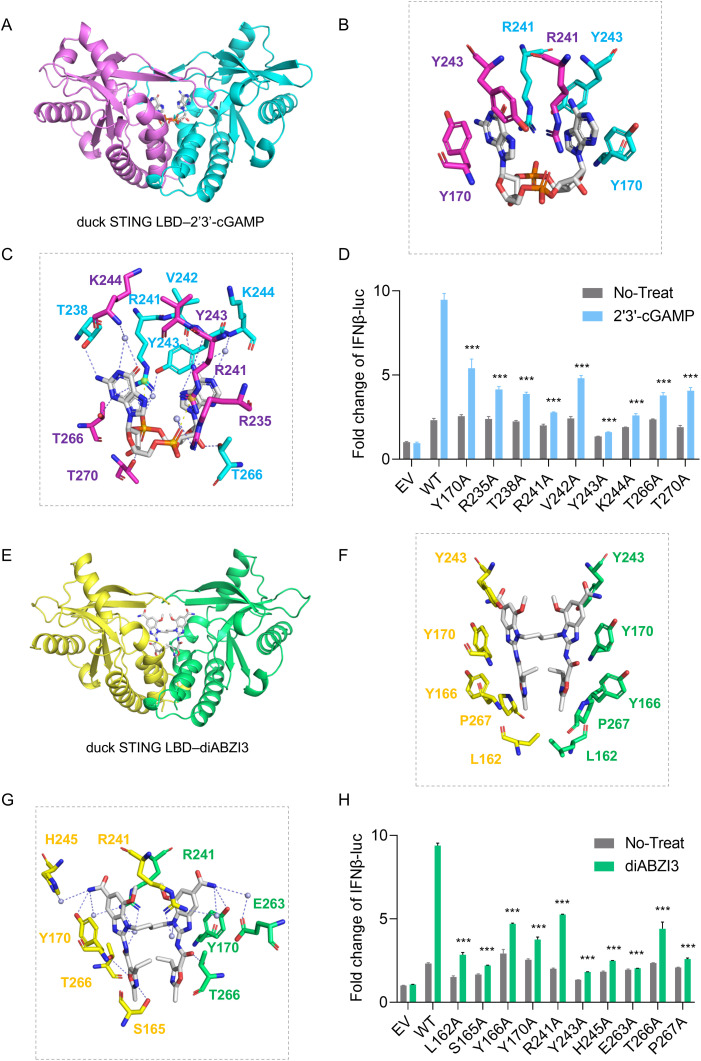
Crystal structures and intermolecular contacts of duck STING LBD bound to 2′3′-cGAMP and diABZI3. **(A)** Crystal structure of 2′3′-cGAMP–bound to duck STING LBD. The symmetrical duck STING LBD dimer was shown in a cartoon representation, with individual monomers colored in magenta and cyan. **(B, C)** Intermolecular contacts in the complex of 2′3′-cGAMP–bound to duck STING LBD, The bound 2′3′-cGAMP was shown in gray color, with the residues of individual STING monomers were shown in magenta and cyan. **(D)** IFN-β luciferase reporter assays in HEK-293T cells expressing duck STING, with or without 2′3′-cGAMP stimulation. All data represented three biological replicates, error bars were ± SD and significance is determined by Students t-test; * *P* < 0.05, ** *P* < 0.01, *** *P* < 0.001. **(E)** Crystal structure of diABZI3 bound to duck STING LBD. The symmetrical duck STING LBD dimer was shown in a cartoon representation, with individual monomers colored in yellow and lime green. **(F, G)** Intermolecular contacts in the complex of diABZI3 bound to duck STING LBD. The bound diAZBI3 was shown in gray color, with the residues of individual STING monomers were shown in yellow and lime green. **(H)** IFN-β luciferase reporter assays in HEK-293T cells expressing duck STING, with or without diABZI3 stimulation. All data represented three biological replicates, error bars were ± SD and significance is determined by Students t-test; * *P* < 0.05, ** *P* < 0.01, *** *P* < 0.001.

Structural analysis of the ligand-binding pocket of duck STING revealed that ligand stabilization was mediated by an integrated network of hydrophobic contacts and polar interactions (**Fig 3A**). For 2′3′-cGAMP, the purine bases were positioned through stacking-like hydrophobic contacts with the aromatic side chains of Y170 and Y243, which help define ligand orientation within the binding pocket (**Fig 3B**). In parallel, the two α-phosphate groups were electrostatically coordinated by salt bridges with the side-chain guanidinium groups of R241 from each protomer, providing strong stabilization of the negatively charged phosphate backbone (**Fig 3B**). At the level of base recognition, T238 and T266 formed direct hydrogen bonds to polar atoms of the ligand via their side-chain hydroxyl groups. Beyond these direct contacts, ligand binding was reinforced by a multilayered hydrogen-bonding network comprising both direct and water-mediated interactions (**Fig 3C**). Above the ligand, K244 contributed to this network primarily through hydrogen bonding via its side-chain amino group. Below the ligand, water-mediated hydrogen bonds linked the side-chain guanidinium group of R235 and the phenolic hydroxyl group of Y243 to the ligand, further stabilizing ligand recognition. Notably, V242 contributed predominantly through its backbone amide N–H, participating in both direct and water-bridged hydrogen bonds, thereby supporting the architecture of the hydrogen-bonding network rather than side-chain-specific recognition. Finally, T270 formed a direct hydrogen bond via its side-chain hydroxyl group with a non-bridging oxygen atom of the phosphate backbone, effectively constraining phosphate geometry and anchoring the cyclic dinucleotide within the pocket (**Fig 3C**). Guided by these structural observations, we generated duck STING mutants targeting key ligand-contacting residues to disrupt the corresponding STING-ligand interactions. Luciferase reporter assays showed that all mutants markedly reduced IFN-β reporter activity upon 2′3′-cGAMP stimulation, confirming the critical role of these residues in 2′3′-cGAMP binding (**Fig 3D**).

The LBD of duck STING primarily engaged the agonist diABZI3 through hydrophobic contacts and hydrogen bonds (**Fig 3E**). Within the binding pocket, diABZI3 established extensive hydrophobic contacts with Leu162 through its aliphatic side chain, with Y170 and Y243 through their aromatic side chains, and with P267 through its cyclic side chain, which together represent major energetic contributors to ligand binding (**Fig 3F**). Polar interactions further refined ligand positioning: S165 and T266 formed direct hydrogen bonds with diABZI3 via their side-chain hydroxyl groups, and the phenolic hydroxyl group of Y170 also participated in direct hydrogen-bond coordination (**Fig 3G**). In addition, a network of water-mediated hydrogen bonds connected diABZI3 to R241 via its side-chain guanidinium group, to H245 via its side-chain imidazole ring, and to E263 via its side-chain carboxylate group, thereby reinforcing ligand anchoring and constraining its orientation within the binding pocket (**Fig 3G**). Based on these interactions, we designed a panel of duck STING mutants (single-letter amino acid notation: L162A, S165A, Y166A, Y170A, R241A, Y243A, H245A, E263A, T266A, and P267A). Luciferase reporter assays demonstrated that all mutations substantially impaired IFN-β promoter activity, validating their role in the binding of diABZI3 (**Fig 3H**).

Collectively, these results show that duck STING recognizes 2′3′-cGAMP and diABZI3 through partially overlapping yet distinct sets of ligand contacting residues. Stable binding of both agonists relies on a shared network of hydrophobic contacts and hydrogen bonds, whereas unique polar interactions underlie specificity and may influence binding orientation and activation potency. Mutagenesis experiments further establish these residues as critical determinants of ligand recognition and downstream activation, providing a molecular framework for understanding ligand specificity in duck STING.

### A novel Z-shaped tetrameric assembly of duck STING

**Fig 4:**
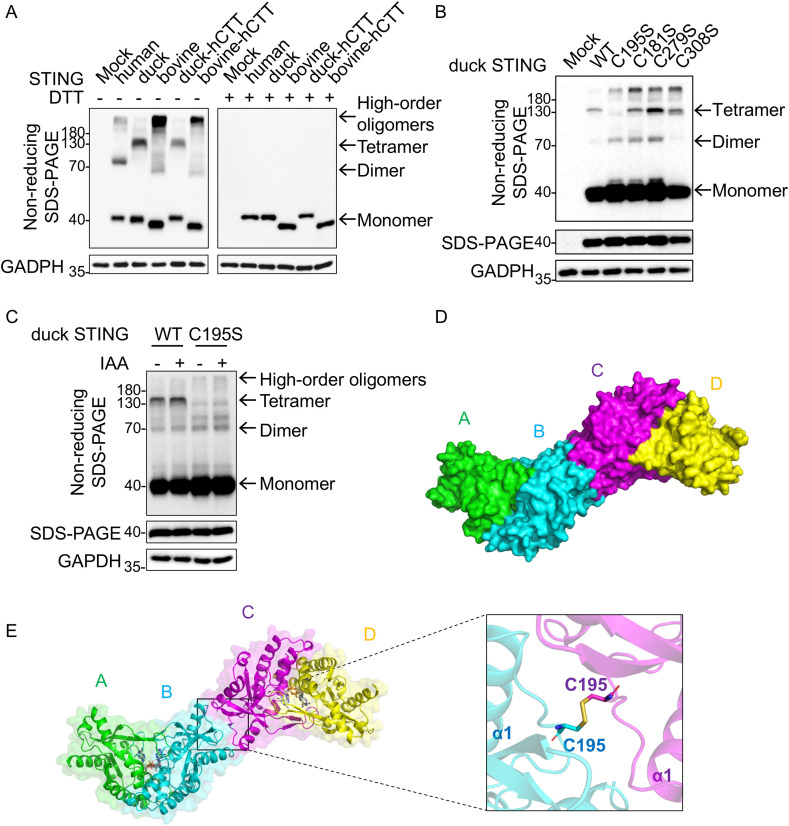
Unique tetrameric assembly of duck STING. **(A)** STING oligomerization detected by SDS-PAGE/Western blotting in HEK-293T cells expressing human STING wild-type (WT), duck STING WT, bovine STING WT, duck-HCTT, and bovine-HCTT under non-reducing (−DTT, left) and reducing (+DTT, right) conditions. The data were representative of three biological replicates. **(B)** STING oligomerization detected by non-reducing SDS-PAGE/Western blotting in HEK-293T cells expressing duck STING WT or the C195S, C181S, C279S, and C308S mutants. The data were representative of three biological replicates. **(C)** Non-reducing SDS-PAGE analysis of HEK-293T cells transfected with wild-type duck STING or the C195S mutant, with lysis performed in the presence or absence of 50 mM iodoacetamide (IAA). **(D)** A Z-shaped tetramer of 2′3′-cGAMP bound to duck STING LBD complex is observed in the crystal lattice, shown in surface representation; protomers A-D were colored green, cyan, magenta, and yellow. **(E)** Tetramer of 2′3′-cGAMP bound to duck STING LBD shown in cartoon representation, the inset illustrates the interprotomer disulfide connection between C195 residues of protomer B and protomer C**.**

STING signaling is tightly regulated by oligomerization [26,36,37]. Because SDS disrupts noncovalent contacts while non-reducing conditions preserve disulfide bonds, non-reducing SDS-PAGE provides a means to detect disulfide-mediated oligomers. We therefore compared disulfide-mediated oligomeric states of human, bovine, and duck STING. In HEK-293T cells expressing full-length STING, non-reducing SDS-PAGE revealed that human STING migrated predominantly as a C148 disulfide-linked dimer, consistent with previous reports [26,38], bovine STING migrated mainly as high-order oligomers (larger than dimers); and duck STING exhibited a distinct tetrameric band not observed in human and bovine (**Fig 4A, left**). Upon treatment with DTT, all oligomeric bands disappeared, confirming that disulfide bonds mediated oligomerization of STING (**Fig 4A, right**). Consistently, non-reducing SDS-PAGE of purified recombinant STING LBDs showed that the duck STING LBD migrated as disulfide-linked oligomers, whereas bovine and human LBDs migrated exclusively as monomers (S3A Fig). To rule out any contribution of the C-terminal tail (CTT) to tetramer formation, we generated a chimeric construct (duck-hCTT) in which the duck CTT was replaced with its human counterpart (S3B Fig). Non-reducing SDS-PAGE analysis revealed that the chimeric STING retained the disulfide-linked tetrameric pattern, demonstrating that tetramerization is mediated by the duck STING LBD (**Fig 4A**). To investigate the basis for the distinct oligomerization properties of duck STING LBD compared with human, bovine STING LBDs, we performed multiple sequence alignment of STING LBDs from duck, human, and bovine, revealing four cysteines unique to duck STING LBD: C181, C195, C279, and C308 (S3C Fig). We then generated full-length STING mutants (C181S, C195S, C279S, C308S) and analyzed their oligomerization under non-reducing conditions. While the C181S, C279S, and C308S mutants retained the tetrameric band, the C195S mutation completely abolished tetramer formation (**Fig 4B**). To test whether the C195-mediated disulfide-linked tetramer forms prior to cell lysis, we added iodoacetamide (IAA) to the lysis buffer of cells expressing duck STING WT or C195S to block free thiols. Addition of IAA did not reduce the abundance of disulfide-linked STING in cells expressing duck STING WT, whereas the C195S mutant remained unable to form tetramer (**Fig 4C**). These results support the conclusion that the C195-mediated disulfide bond forms intracellularly before lysis rather than arising during sample processing. In agreement, purified duck STING LBD carrying C195S mutant protein migrated exclusively as a monomer, eliminating disulfide-linked bands (S3D **and** S3E Fig). These results identify C195 as the key cysteine required for formation of the disulfide-linked tetrameric species of duck STING.

To rationalize these findings at atomic resolution, we examined the crystal lattice of the duck STING–2′3′-cGAMP complex and identified a previously unreported “Z-shaped” tetramer. The protomers, designated as A, B, C, and D from left to right (**Fig 4D**), assembled when an additional interface between protomers B and C brings two V-shaped dimers into mutually inverted orientations and links them edge-to-edge, forming the Z-shaped tetrameric arrangement. Notably, the interface between protomers B and C buried about 1950 Å² of surface area, and within this interface, the C195 residues from each protomer formed a disulfide bond (**Fig 4E**). This Z-shaped tetrameric arrangement in duck STING LBD was markedly different from the oligomerization mode reported in other species STING LBD (S4A**-**S4G Fig). In published ligand-bound structures from human STING [8,26,30] (PDB: 4KSY, 4LOH, 4F5D, 6CFF; S4A**-**S4D Fig), mouse STING [23] (PDB: 4LOJ; S4E Fig), anemone STING [39] (PDB: 5CFQ; S4F Fig), bacterial STING [40] (PDB: 7EBD; S4G Fig), STING instead formed extended side-by-side linear polymers via inter-dimer salt bridges. Recent cryo-EM studies of full-length STING further emphasize the structural difference [24,27,41,42]. In the activated, ligand-bound conformation, STING protomers assembled into a side-by-side filamentous arrangement that facilitates TBK1 and IRF3 recruitment (PDB: 8IK3; S4H Fig). By contrast, in the apo, self-inhibited conformation, STING adopted a head-to-head packing that sterically hinders downstream factor binding and preserves the inactive state (PDB: 8IK0; S4I Fig).

Sequence alignment of STING proteins across Insecta, Actinopterygii, Amphibia, Aves, and Mammalia revealed that C195 was conserved exclusively within a subset of Aves, predominantly waterfowl, including duck (*Anas platyrhynchos*), swan goose (*Anser cygnoides*) and black swan (*Cygnus atratus*), but absent in mammals and other vertebrate classes (S1A Fig). Across seven waterfowl species, C195 is highly conserved (S3F Fig and S3 Table), suggesting a lineage-specific evolutionary adaptation.

### The Z-shaped tetrameric assembly mediates the autoinhibition of duck STING

Building on the structural and biochemical evidence for a C195-dependent tetrameric assembly, we next examined the functional contribution of C195 to the oligomerization behavior of full-length duck STING. To directly assess the contribution of residue C195 to oligomerization of full-length duck STING, we analyzed STING assemblies using native gel electrophoresis. HEK-293T stable cell lines expressing duck STING WT or C195S mutant were generated (S5A Fig). Under unstimulated conditions, the STING C195S mutant exhibited significantly enhanced oligomerization compared with STING WT. Upon stimulation with either 2′3′-cGAMP or diABZI3, both WT and C195S STING formed high-order oligomers; however, the abundance and extent of oligomer formation were consistently greater for C195S mutant than for WT under both stimulation conditions (S5B Fig). These results indicate that full-length duck STING exists in multiple assembly states and that C195 restrains its oligomerization both at baseline and after ligand stimulation, and the C195-dependent tetramer likely represents one autoinhibitory assembly state under resting conditions.

**Fig 5:**
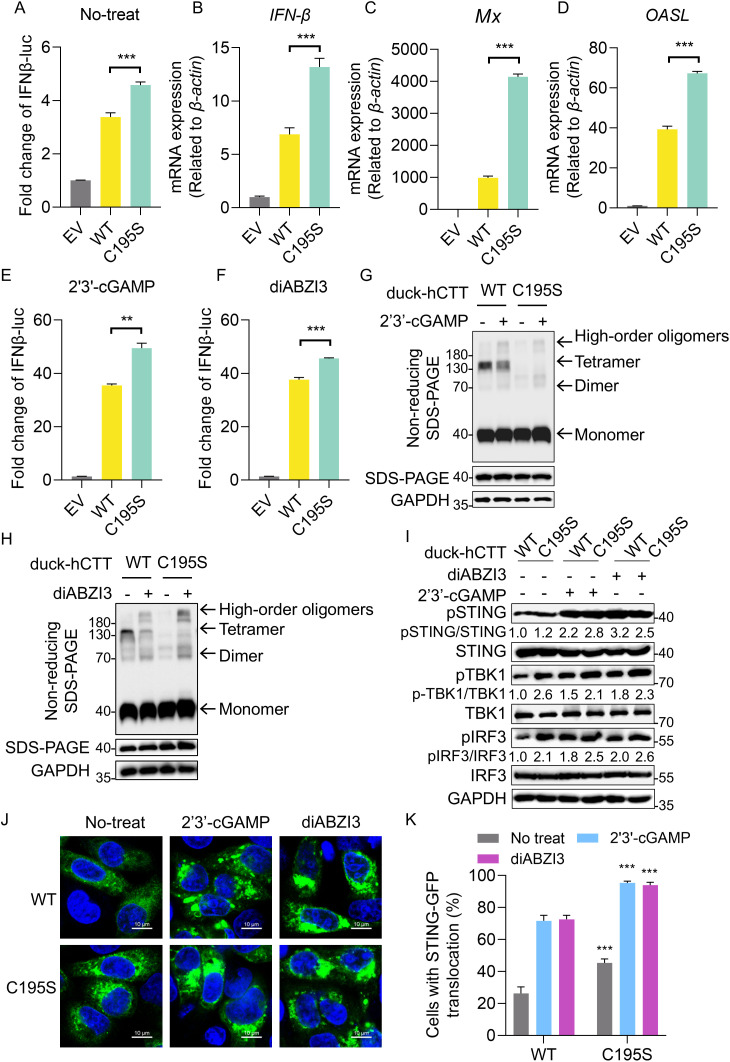
C195 is required for the autoinhibition of duck STING. **(A)** IFN-β luciferase reporter assays in HEK-293T cells expressing duck-hCTT STING (WT) or the C195S mutant under untreated. All data represented three biological replicates, error bars were ± SD and significance is determined by Students t-test; * *P* < 0.05, ** *P* < 0.01, *** *P* < 0.001. **(B-D)** The qRT–PCR analysis of *IFN-β* **(B)**, *Mx* (**C**) and *OASL* (**D**) mRNA levels in DEFs transfected with duck STING (WT) or the C195S mutant, values were normalized to β-actin. All data represented three biological replicates, error bars were ± SD and significance is determined by Students t-test; * *P* < 0.05, ** *P* < 0.01, *** *P* < 0.001. **(E-F)** IFN-β luciferase reporter assays in HEK-293T cells expressing duck-hCTT STING (WT) or the C195S mutant under 2′3′-cGAMP–treated **(E)**, and diABZI3-treated (**F**) conditions. All data represented three biological replicates, error bars were ± SD and significance is determined by Students t-test; * *P* < 0.05, ** *P* < 0.01, *** *P* < 0.001. **(G)** Non-reducing SDS–PAGE/Western blotting of STING oligomerization in HEK-293T cells expressing duck-hCTT WT and C195S in the absence or presence of 2′3′-cGAMP. **(H)** Non-reducing SDS–PAGE/Western blotting of STING oligomerization in HEK-293T cells expressing duck-hCTT WT and C195S in the absence or presence of diABZI3. **(I)** Effect of the duck STING C195S mutation on phosphorylation in the STING pathway by Western blotting analysis. **(J-K)** Immunofluorescence analysis of STING C195S mutation on the formation of STING foci in cGAS-knockdown HeLa cells under untreated, cGAMP-treated, or diABZI3-treated conditions. Scale bars, 10 μm. Representative confocal images for each condition were shown; the quantification in panel K is based on at least 200 cells. All data represented three biological replicates, error bars were ± SD and significance is determined by Students t-test; * *P* < 0.05, ** *P* < 0.01, *** *P* < 0.001.

Species-specific differences in signal transduction within the cGAS–STING pathway have been reported between avian and mammalian hosts [32]. Within the CTT of human STING, the PLPLRT/SD motif [21], which recruits and activates TBK1, and the *p*L*x*IS motif [43] (*p* represents the hydrophilic residue, *x* represents any residue, and S represents the phosphorylation site), which recruits and activates IRF3, differ at the corresponding positions in duck STING (S6A Fig). Likewise, P361 in human STING, a residue critical for IRF3 binding [22], is replaced by L364 in duck STING, highlighted with a yellow box (S6A Fig). To enhance the relatively weak signaling output of duck STING in human cells, we used a chimeric construct (duck-hCTT) in place of WT duck STING, and performed IFN-β luciferase reporter and phosphorylation assays in HEK-293T cells (S6B Fig). Importantly, the duck-hCTT construct preserved the formation of the duck-specific tetramer (**Fig 4A**). In IFN-β luciferase assays, the duck-hCTT C195S mutant exhibited significantly higher activity than wild-type duck-hCTT (**Fig 5A**). In a duck-origin context, non-reducing SDS-PAGE in duck embryo fibroblasts (DEFs) confirmed that C195S abolished the tetrameric band (S6C Fig), while qRT-PCR revealed marked upregulation of *IFN-β* and interferon-stimulated genes (ISGs), *Mx* and *OASL,* relative to WT (**Fig 5B-5D**). Similarly, upon stimulation with 2′3′-cGAMP or diABZI3, the C195S mutant produced higher IFN-β reporter activity than WT (**Fig 5E and 5F**). Notably, agonist stimulation reorganized the assembly profile of WT duck-hCTT, increasing dimer and high-order oligomer bands while reducing tetramer bands (**Figs 5G-5H and** S6D). To further substantiate this phenotype, we quantified phosphorylation of STING-pathway nodes and visualized STING puncta by confocal microscopy in HEK-293T cells. In cells transfected with the C195S mutant, we observed elevated basal phosphorylation of STING, TBK1 and IRF3, as well as increased puncta formation, both in the absence and presence of 2′3′-cGAMP or diABZI3 (**Fig 5I-5K**). Taken together, these data indicate that the C195-driven Z-shaped tetramer contributes to autoinhibition of duck STING. Disruption of this C195-centered interface, either by mutation or through ligand-associated remodeling of STING assemblies, relieves this inhibitory restraint.

### Noncovalent interactions cooperatively stabilize the Z-shaped tetramer of duck STING

**Fig 6:**
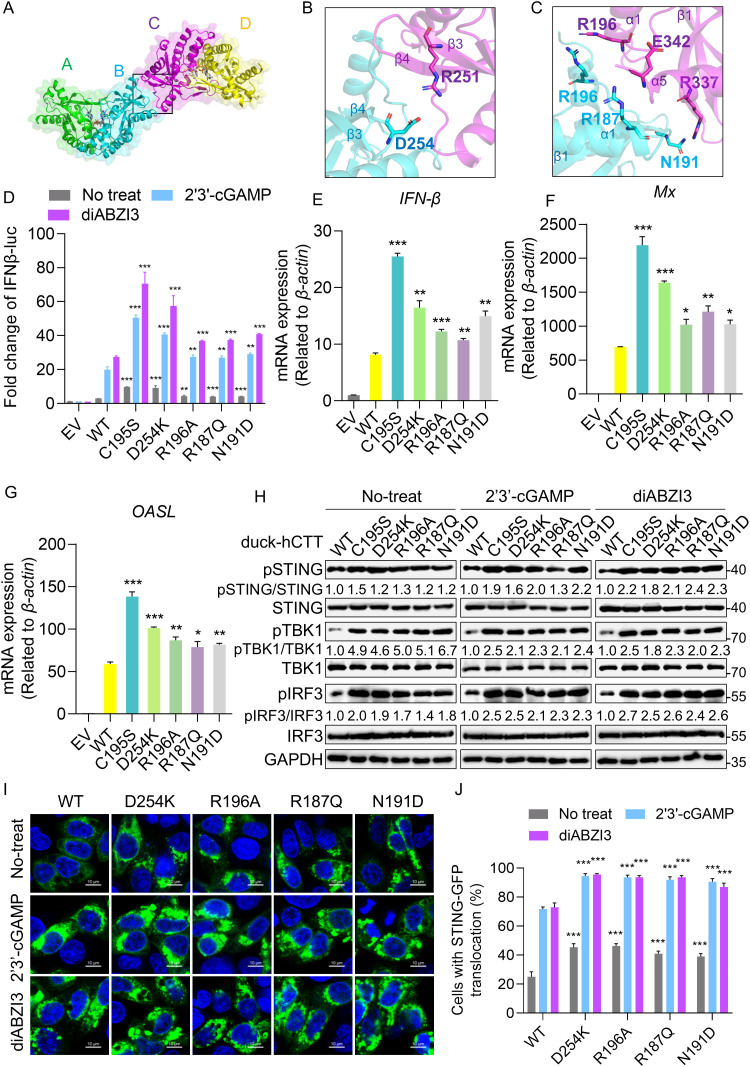
Mutational dissection of the cooperative autoinhibitory network in duck STING. **(A-C)** Tetrameric assembly of duck STING LBD in the crystal lattice and shown in cartoon **(A)**. Insets showed interfacial contacts between protomers B and C, including salt-bridge interactions (**B**) and hydrogen-bond interactions (**C**) of duck STING LBD crystal structures. **(D)** IFN-β luciferase reporter assays in HEK-293T cells expressing duck-hCTT STING WT or mutants under untreated, 2′3′-cGAMP treated, and diABZI3 treated conditions. All data represented three biological replicates, error bars were ± SD and significance is determined by Students t-test; * *P* < 0.05, ** *P* < 0.01, *** *P* < 0.001. **(E-G)** The qRT–PCR analysis of *IFN-β* **(E)**, *Mx* (**F**) and *OASL* (**G**) mRNA levels in DEFs transfected with duck STING WT or mutants, values were normalized to β-actin. All data represented three biological replicates, error bars were ± SD and significance is determined by Students t-test; * *P* < 0.05, ** *P* < 0.01, *** *P* < 0.001. **(H)** Effect of the duck STING mutations on phosphorylation in the STING pathway by Western blotting analysis. **(I-J)** Immunofluorescence analysis of STING mutations on the formation of STING foci in cGAS-knockdown HeLa cells under untreated, cGAMP-treated, or diABZI3-treated conditions. Scale bars, 10 μM. Representative confocal images for each condition were shown; the quantification in panel J is based on at least 200 cells. All data represented three biological replicates, error bars were ± SD and significance is determined by Students t-test; * *P* < 0.05, ** *P* < 0.01, *** *P* < 0.001.

While the C195–C195 disulfide is the key determinant of tetramer formation in duck STING, we next characterized the noncovalent network that reinforces the interface between protomers B and C. In addition to the C195-C195 disulfide, the buried surface contained a set of symmetry-related salt bridges and hydrogen bonds that stabilize the Z-shaped autoinhibited assembly (**Fig 6A**). At the LBD β3–β4 turn, Asp254 (D254) formed a salt bridge with Arg251 (R251) on β3 (**Fig 6B**). Arg187 (R187) on helix α1 formed a hydrogen bond with Glu342 (E342) on helix α4. Arg196 (R196) in the α1–β1 loop of protomer B formed a hydrogen bond with Arg196 in protomer C. Additionally, Asn191 (N191) in the α1–β1 loop hydrogen bound with Arg337 (R337) in the helix α4 (**Fig 6C**). These contacts laterally interlock protomers B and C, reinforcing the tetrameric interface.

To assess functional relevance of these interactions, we generated interface mutants and examined STING pathway activation. Mutants D254K, R196A, R187Q, and N191D displayed ligand-independent activation relative to WT duck-hCTT in IFN-β luciferase assays (**Fig 6D**). In DEFs, these substitutions increased mRNA levels of *IFN-β* and ISGs, *Mx* and *OASL* (**Fig 6E-6G**). Consistently, with or without 2′3′-cGAMP or diABZI3 stimulation, these mutants displayed enhanced signaling, including higher IFN-β reporter activity, enhanced STING, TBK1 and IRF3 phosphorylation, and increased STING puncta formation (**Fig 6H-6J**).

To further assess the evolutionary conservation of residues comprising the Z-shaped tetrameric interface, we performed comparative sequence analyses across the lineages of Aves, Mammalia, Insecta, Actinopterygii and Amphibia (S4 Table). The β3–β4 salt-bridge pair D254 and its interacting partner R251 was strictly conserved in waterfowl. In several non-waterfowl avian species, these positions remained relatively conserved at the amino acid level, whereas in mammals they underwent coordinated substitutions that disrupt the charge complementarity required for salt-bridge formation. By contrast, residues comprising the polar and hydrogen-bonding network of the Z-shaped interface, including R187–E342, N191–R337, and the symmetric R196–R196 contact, exhibited a more pronounced waterfowl-restricted conservation pattern. Within waterfowl, these residues were invariant or only conservatively substituted, preserving their chemical properties and interaction potential. Outside the waterfowl lineage, however, the corresponding interaction pairs were frequently not co-retained, consistent with erosion of the coordinated polar interaction network. Notably, R196 was invariantly conserved as arginine across all examined waterfowl species, supporting its role in forming the symmetric R196–R196 polar contact at the tetrameric interface. In contrast, the corresponding position in mammals was most often occupied by shorter, nonbasic residues (such as A, L, T, or I), which are incompatible with formation of this interaction.

**Fig 7:**
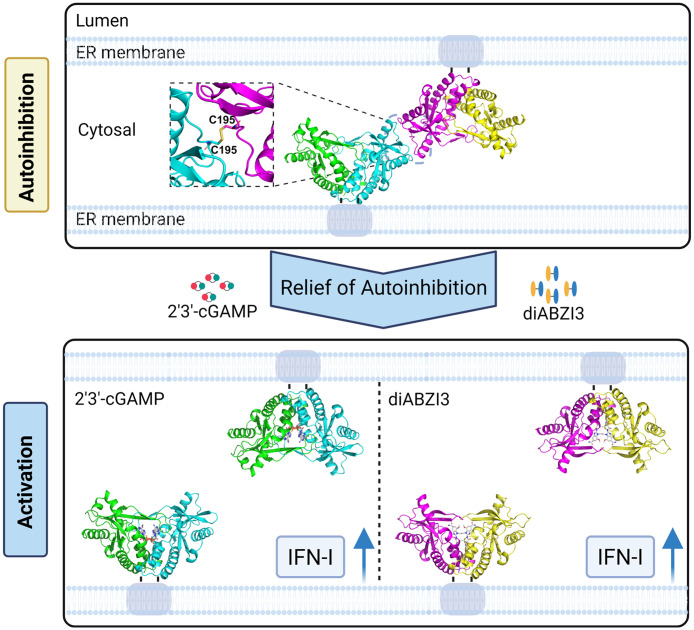
Z-Shaped tetrameric assembly mediated autoinhibition and agonist induced activation of duck STING. In the inactive state, duck STING assembles into a unique Z-shaped tetramer at the cytosolic leaflet of the endoplasmic reticulum (ER) membrane, stabilized by a C195–C195 disulfide bond and supporting polar interactions. This oligomeric assembly maintains STING in an autoinhibited conformation and suppresses type I interferon (IFN-I) signaling. Upon binding to 2′3′-cGAMP or diABZI3, the tetrameric interface is disrupted, allowing STING to undergo conformational unlocking, reorganize into an active oligomeric state, and induce IFN-I expression. Created in BioRender. Huang, **X.** (2026) https://BioRender.com/p9mewyt.

Collectively, these results indicate that the D254-R251 salt bridge, R187-E342 hydrogen bond, R196-R196 polar contact, and N191-R337 hydrogen bond cooperate with the C195 disulfide to form a composite interface network that stabilizes the autoinhibitory tetramer of duck STING. Disruption of key interactions within this network lowers the autoinhibitory threshold, allowing ligand-independent activation and amplifying agonist-induced STING signaling (**Fig 7**).

## Discussion

STING is a central player in the innate immune response, coupling the sensing of aberrant cytosolic DNA to interferon production and playing a critical role in host defense against infection and cancer [44,45]. Pathologic STING activation is associated with multiple autoinflammatory syndromes and has emerged as a key target in cancer immunotherapy [46,47]. Previous studies reported that apo-STING adopts a head-to-head autoinhibited bilayer assembly, whereas ligand-bound STING forms a closed dimer that further polymerizes side-by-side in the activated state [24]. However, evolutionary and structural insights into avian STING have remained limited.

Here we determined high-resolution crystal structures of duck and bovine STING–2′3′-cGAMP complexes, as well as duck, bovine, and human STING–diABZI3 complexes. Integrating biochemical and cell-based analyses, we observed that duck STING assembled into a Z-shaped tetramer stabilized by an interfacial C195-C195 disulfide and a cooperative polar network. This tetrameric assembly is distinct from the side-by-side polymers of activated STING and the head-to-head assembly of apo-STING, representing a previously unrecognized regulatory mechanism of STING.

In the crystal structure of duck STING LBD–2′3′-cGAMP, the tetramer interface is mediated by the C195-C195 disulfide and a network of symmetry-related salt bridges and hydrogen bonds (D254-R251, R187-E342, R196-R196, N191-R337) that laterally interlock adjacent protomers. Although a crystal structure of duck apo-STING was not obtained, non-reducing SDS-PAGE showed that duck apo-STING can form and migrate as disulfide-linked tetrameric species, whereas human STING migrates mainly as disulfide-linked dimers and bovine STING as high-order disulfide-linked oligomers, highlighting species-specific assembly characteristics. Consistent with prior work, oligomerization of human STING is primarily mediated by cysteine 148 (C148) within the linker region connecting the ligand-binding domain (LBD) to the transmembrane domain (TMD) [26]. In contrast, to explain the robust high-order oligomerization observed for bovine STING, we compared full length sequences across species and found that bovine STING uniquely contains multiple cysteine residues within its TMD (C71, C90, C95, C103, and C107) that are absent from both human and duck STING (S1B Fig). Functional analyses further demonstrated that mutation of these TMD cysteines markedly reduced high-order oligomer formation in bovine STING, with the C107S single mutant and the C103/C107S double mutant nearly abolishing oligomerization (S6E Fig). These findings indicate that STING oligomerization is controlled by distinct, species-specific molecular determinants, with duck STING relying on a C195-centered LBD interface and bovine STING predominantly governed by TMD-encoded cysteine chemistry.

The C195S mutation abolishes the disulfide-linked tetrameric species and markedly enhances phosphorylation of STING, TBK1, and IRF3, increases STING puncta formation, and elevates IFN-β reporter activity. Upon stimulation with 2′3′-cGAMP or diABZI3, the tetramer is strongly depleted, accompanied by increased disulfide-linked dimers and high-order oligomers. In DEFs, the C195S mutation further elevates mRNA levels of *IFN-β* and downstream ISGs, *Mx* and *OASL*. Notably, native analysis of full-length duck STING oligomerization further supports this functional model, showing that duck STING exists in multiple assembly states and that disruption of C195 enhances oligomerization under both resting and ligand-stimulated conditions. Together, these findings indicate that the C195-dependent tetramer restrains excessive high-order assembly of duck STING and support a C195-mediated autoinhibitory model.

Notably, our data further indicate that this autoinhibitory interface is stabilized not only by the C195-C195 disulfide bond, but also by a surrounding network of noncovalent interactions. Mutations such as D254K, R196A, R187Q, and N191D, which disrupt key noncovalent contacts within the tetramer interface, are likewise sufficient to trigger ligand-independent activation. These findings suggest that the Z-shaped tetramer is maintained by a composite inter-protomer interface in which the disulfide bond cooperates with surrounding salt bridges and hydrogen bonds. This multi-point architecture implies that release of the tetrameric constraint is unlikely to require rupture of a single rigid covalent linkage. Rather, partial destabilization of this cooperative interface may be sufficient to lower the energetic barrier for tetramer remodeling or dissociation.

Importantly, this autoinhibitory model is conceptually consistent with established mechanisms of ligand-dependent STING activation. We propose that the C195-dependent Z-shaped assembly serves as a threshold-setting autoinhibitory state under resting conditions. Within this framework, ligand binding does not simply disrupt an isolated disulfide bond; instead, it drives the canonical conformational rearrangements of STING, including the ~ 180° rotation of the LBD relative to the TMD and reorganization of oligomerization interfaces. These coordinated changes collectively weaken and remodel the C195-centered autoinhibitory interface, thereby releasing this inhibitory constraint. STING can then proceed along the established activation trajectory and assemble into signaling-competent high-order oligomers. Consistent with this model, stimulation of WT duck STING with either 2′3′-cGAMP or diABZI3 triggers a pronounced shift in the oligomeric landscape, with depletion of the disulfide-linked tetramer and concomitant accumulation of dimeric and high-order oligomeric species. Together, these observations suggest that remodeling and depletion of the autoinhibitory tetramer accompany STING activation and reflect release of an upstream autoinhibitory constraint during the transition to signaling-competent assemblies.

Consistent with this functional model, comparative sequence analyses reveal that the Z-shaped tetramer interface is supported by a set of residues that are cooperatively conserved within waterfowl (S4 Table). Beyond the C195–C195 disulfide, multiple residues contributing to the noncovalent interaction network are collectively preserved in waterfowl but diverge to varying extents in other lineages, supporting the notion that the Z-shaped autoinhibitory tetramer represents a composite regulatory module that co-evolved and has been selectively maintained in waterfowl. In contrast, the head-to-head autoinhibitory arrangement described for chicken apo-STING is not conserved in waterfowl (S5 Table). Although non-waterfowl birds and mammals largely retain the hydrophobic features required for the canonical head-to-head interface, waterfowl exhibit coordinated substitutions at determinant positions and surrounding interface residues that are predicted to weaken a classical “chicken-like” head-to-head arrangement. Together, these patterns support a model in which waterfowl STING has evolved a distinct autoinhibitory steady-state configuration centered on the Z-shaped tetramer.

Notably, our structural observations derive primarily from the arrangement of LBD protomers within the crystal lattice. Future studies using full-length duck STING by cryo-EM combined with cellular imaging will be necessary to quantify tetramer abundance and dynamics under physiological conditions.

In summary, we identify a previously unrecognized Z-shaped tetrameric assembly of duck STING, formed through a C195-C195 disulfide linkage between two STING dimers and further stabilized by a cooperative network of noncovalent interfacial interactions, and that likely contributes to autoinhibitory control of duck STING. We further define species-dependent features of dimer closure and map key determinants governing recognition of 2′3′-cGAMP and diABZI3. This work fills a critical structural gap for waterfowl STING, particularly in duck, expands the structural landscape of distinct STING assembly, and provides a comparative structural framework for understanding species-specific regulation of STING-mediated signaling.

## Materials and methods

### Cell lines

HEK-293T and HeLa cGAS-knockdown cells were cultured in DMEM (MACGENE, CM10013) with 10% fetal bovine serum (FBS) (PlantChemMed, PC00001), and Duck Embryonic Fibroblasts (DEFs) were cultured in complete medium (Ubigene, YM-P-369); all cells were maintained at 37 °C with 5% CO_2_.

### Regents and antibodies

2′3′-cGAMP (# HY-100564) was purchased from MCE. diABZI3 (# S8796) was purchased from Selleck. The following antibodies were used in this study: Primary antibodies were rabbit anti HA-Tag monoclonal antibody (mAb) (CST, # 3724S), rabbit anti STING (D2P2F) mAb (CST, # 13647), rabbit anti phospho-STING (Ser366) (E9A9K) mAb (CST, # 50907), TBK1/NAK (D1B4) Rabbit mAb (CST, # 3504), rabbit anti phospho-TBK1/NAK (Ser172) (D52C2) mAb (CST, # 5483), rabbit anti-IRF3 (phosphoS386) antibody (Abcam, #EPR2346), rabbit anti IRF3 (D83B9) mAb (CST, # 4302), rabbit anti GAPDH mAb (Proteintech, # 10494–1-AP). Secondary antibody was anti Rabbit IgG HRP-Linked antibody (CST, # 7074).

### Protein expression and purification

The cDNA fragments encoding the ligand-binding domain (LBD) of duck STING (residues 158–344) and human STING (residues 155–341) were cloned into pET28a-SUMO. In this vector, the target protein was linked to an N-terminal His6–SUMO tag that can be removed via a ULP1 (ubiquitin-like protease 1) cleavage site positioned between the tag and the target sequence. The cDNA encoding bovine STING LBD (residues 158–344) was cloned into pET-30a. The target protein was expressed in soluble form in *E. coli* BL21(DE3) following induction. Briefly, positive BL21(DE3) clones were grown in LB supplemented with kanamycin (30 μg/mL) to an OD600 of 1.0, induced with 0.4 mM IPTG, and expressed at 16 °C for 20 h. The cells were resuspended in lysis buffer (50 mM Tris-HCl pH 8.0, 300 mM NaCl) and lysed by sonication.

For duck and human STING LBD proteins, clarified supernatants were purified by Ni-NTA affinity chromatography, followed by ULP1 digestion to remove the His6–SUMO tag. Samples were dialyzed against 20 mM Tris-HCl pH 7.5, 150 mM NaCl, passed over Ni-NTA again to remove the His6–SUMO tag, and further purified by gel-filtration chromatography on Superose 6 Increase 10/300 GL column in 20 mM Tris-HCl pH 7.5, 150 mM NaCl. Final concentrations for duck and human STING LBD proteins were ~15 mg/mL. The duck C195S LBD mutant protein was purified using the same protocol as the duck STING LBD protein. For bovine STING LBD protein, the Ni-NTA purified fusion protein was first polished by a HiLoad 16/600 Superose 200 pg column to remove contaminants; peak-apex fractions were then subjected to a second purified step on Superose 6 Increase 10/300 GL column in 20 mM Tris-HCl pH 7.5, 150 mM NaCl. The final bovine STING LBD protein concentration was 15 mg/mL. To improve sample homogeneity and minimize nonspecific cysteine oxidation, 5 mM DTT was included during the protein concentration step prior to size-exclusion chromatography.

### Crystallization, data collection, structure determination, and refinement

Before crystallization screening, duck or bovine STING LBD was mixed with 2′3′-cGAMP at a 1:1.2 and molar ratio, respectively, and incubated for 2 h on ice. The incubation mixture was further purified by gel-filtration chromatography on Superose 6 Increase 10/300 GL column in 20 mM Tris-HCl pH 7.5, 150 mM NaCl, peak-apex fractions corresponding to the complex were collected and concentrated to 15 mg/mL for crystallization screening. Initial crystallization screening was performed at 4 °C using the sitting-drop vapor diffusion method, and crystal quality was optimized by hanging-drop vapor diffusion. Crystals of the duck STING LBD–2′3′-cGAMP complex grew in 1.0 M ammonium sulfate, 0.1 M Bis-Tris (pH 5.5), and 1% (w/v) polyethylene glycol 3350. Crystals of the bovine STING LBD–2′3′-cGAMP complex formed in 1.5 M ammonium chloride and 0.1 M sodium acetate trihydrate (pH 4.6).

For crystallization of STING LBD with diABZI3, duck, bovine or human STING LBD was mixed with diABZI3 at a 1:2 molar ratio, respectively, and incubated for 2 h on ice. The incubation mixture was further purified by gel-filtration chromatography on Superose 6 Increase 10/300 GL column in 20 mM Tris-HCl pH 7.5, 150 mM NaCl, peak-apex fractions corresponding to the complex were collected and concentrated to 15 mg/mL for crystallization screening. Initial crystallization screening was performed at 4 °C using the sitting-drop vapor diffusion method, and crystal quality was optimized by hanging-drop vapor diffusion. Crystals of the duck STING LBD–diABZI3 complex grew in 0.05 M Cesium chloride, 0.1 M MES monohydrate pH 6.5, 30%v/v Jeffamine M-600. Crystals of the bovine STING LBD–diABZI3 complex formed in 0.1M HEPES sodium pH 7.5, 0.8 M Sodium phosphate monobasic monohydrate, 0.8 M Potassium phosphate monobasic. Crystals of the human STING LBD–diABZI3 complex formed in 1 M Ammounium sulfate, 0.1 M Bis-TRIS pH 5.5, 1% PEG3350.

The five complex crystals were protected with 18% (v/v) glycerol or PEG and flash-frozen in liquid nitrogen prior to diffraction and data collection. Diffraction data were collected at beamline BL02U1 of the Shanghai Synchrotron Radiation Facility (SSRF). The data were processed with XDS and scaled using SCALA in the CCP4 package. Structures of the duck and bovine STING LBDs were determined by molecular replacement (MR) using SWISS-MODEL–generated homology models as search templates. Human STING LBD bound to diABZI3 complex structures were solved by molecular replacement using the human STING LBD with diABZI agonist 15 structure (PDB ID: 8STH) as the search model. Manual model building and refinement were performed with Coot and PHENIX, respectively. The final structural models have been deposited in the Protein Data Bank with the accession codes 9WH7, 9WH8, 9WH9, 9WHA and 9WHF. Data collection and refinement statistics are summarized in S2 Table. All structural figures were generated with PyMOL (http://www.pymol.org/).

### Binding assay by isothermal titration calorimetry

Dissociation constants (K_D_) for the binding of duck, bovine, and human STING LBDs to 2′3′-cGAMP were determined by isothermal titration calorimetry (ITC). Protein samples were dialyzed overnight at 4 °C against working buffer (20 mM HEPES pH 7.5, 150 mM NaCl) and both proteins samples and 2′3′-cGAMP were diluted in the same buffer. Specifically, 19 injections (2 μL each) of 250 μM 2′3′-cGAMP were titrated into 300 μL of duck STING LBD or bovine STING LBD (40 μM) or human STING LBD (50 μM). Raw ITC data were processed with MicroCal Analyse and fit to a single-site binding model.

### Luciferase reporter assay

HEK-293T cells were seeded into 24-well plates and, at ~80% confluence, co-transfected using Lipofectamine 2000 (Thermo, #11668019) with a STING expression plasmid, an IFN-β promoter–driven firefly luciferase reporter plasmid, and the pRL-TK Renilla luciferase internal control plasmid. At 24 h post-transfection, cells were stimulated for 6 h with 2′3′-cGAMP (70 μM) or diABZI3 (15 μM). Firefly and Renilla activities were quantified with the Dual-Luciferase Reporter Assay System (Promega) according to the manufacturer’s instructions. Firefly signals were normalized to Renilla and reported as relative luciferase activity (fold change versus mock-treated, untransfected controls).

### Confocal microscopy

HeLa cGAS-knockdown cells were cultured on glass-bottom dishes and transfected with encoding wild-type or mutants full-length EGFP-STING plasmid. At 18 h post-transfection, cells were left untreated or stimulated with 2′3′-cGAMP (70 μM) or diABZI3 (15 μM) for 6 h. Cells were fixed in 4% paraformaldehyde for 30 min, nuclei were counterstained with DAPI for 5 min, and images were acquired on a Nikon A1 laser-scanning confocal microscope and processed in NIS-Elements AR.

### Quantitative real-time PCR (qRT-PCR) analysis

Duck Embryonic Fibroblasts (DEFs) were seeded into 24-well plates and, at ~80% confluence, the cells were transfected with wild-type or mutants duck STING plasmid. After 24 hours, the total RNA was isolated from DEFs using TRIzol (Life Technologies) according to the manufacturer’s instructions. First-strand cDNA was synthesized with the HiScript II Q RT SuperMix kit (Vazyme, #R223-01) using oligo(dT) primers. qPCR was performed on a LightCycler 480 II (Roche) with Taq Pro Universal SYBR qPCR Master Mix; each sample was run in triplicate. Primer pairs were as follows: duck *IFN-β* (F) 5′-TCTACAGAGCCTTGCCTGCAT-3′, (R) 5′-TGTCGGTGTCCAAAAGGATGT-3′; duck *Mx* (F) 5′-TGCTGTCCTTCATGACTTCG-3′, (R) 5′-GCTTTGCTGAGCCGATTAAC-3′; duck *OASL* (F) 5′-TCTTCCTCAGCTGCTTCTCC-3′, (R) 5′-ACTTCGATGGACTCGCTGTT-3′; duck *β-actin* (F) 5′-GATCACAGCCCTGGCACC -3′, (R) 5′-CGGATTCATCATACTCCTGCTT -3′. Relative mRNA levels were quantified using the 2^ ^− ΔΔCt^ method and normalized to *β-actin*.

### Non-reducing SDS-PAGE

Under non-reducing conditions, cell samples were lysed in IP buffer containing Triton X-100 and NP-40, clarified by centrifugation at 15,000 rpm for 15 min at 4 °C, and the resulting supernatants were mixed with SDS sample buffer lacking DTT before separation on 10% SDS-PAGE for downstream analyses. Purified proteins were incubated at 4 °C for 1 h either untreated or in the presence of 2′3′-cGAMP or diABZI3, followed by addition of SDS sample buffer lacking DTT; 10 μg protein per lane was loaded, and gels were stained with Coomassie Brilliant Blue.

### Western blotting

Cell lysates were separated on SDS-polyacrylamide gel electrophoresis (SDS-PAGE) or non-reducing SDS PAGE and transferred to nitrocellulose membranes using a wet-transfer system. For non-reducing SDS-PAGE, membranes were probed with an anti-HA primary antibody. To assess STING-pathway phosphorylation, HEK-293T cells were transfected with wild-type or mutant STING constructs and left untreated or stimulated with 2′3′-cGAMP or diABZI3. Phosphorylated and total STING, TBK1 and IRF3 were detected by immunoblotting with phospho-specific and total antibodies, with GAPDH serving as the loading control.

### Lentivirus packaging and transduction

To generate HEK-293T cell lines stably expressing duck STING wild-type (WT) or the C195S mutant, duck WT-STING-HA and C195S-STING-HA were cloned into the pNL-GFP vector. Lentiviral particles were produced by co-transfecting HEK-293T cells [48] with pNL-STING(WT) or pNL-STING(C195S), psPAX2 and pMD2.G at a plasmid ratio of 1:1:1. Lentiviral supernatants were collected and used to transduce HEK-293T cells in the presence of 8 μg/mL polybrene, followed by centrifugation at 37°C and 2,200 rpm for 2 h. The cells were then cultured at 37°C with 5% CO₂ for an additional 24 h before use in subsequent experiments.

### Native gel analyses of STING oligomerization

HEK-293T cells stably expressing duck WT STING or C195S STING were seeded in 24-well plates. Cells were stimulated with 2′3′-cGAMP or diABZI3 for 12 h, then collected and resuspended in NP-40 lysis buffer (N8032, Solarbio) and incubated on ice for 1 h. The lysates were subjected to micro-sonication, followed by centrifugation at 12,000 rpm for 10 min. The insoluble pellets were discarded, and the supernatants were resuspended in native-PAGE loading buffer (P1017, Solarbio) for subsequent native PAGE analysis.

### Statistical analysis

All statistical analyses were performed using GraphPad Prism 8.3.0. Data are presented as mean ± SD at least three independent experiments. Statistical significance was assessed with Student’s t-test, with *P* < 0.05 considered significant. * *P* < 0.05; ** *P* < 0.01; *** *P* < 0.001.

## Supporting information

S1 Fig Phylogenetic analysis and sequence alignment of STING. (**A**) Phylogenetic tree of STING sequences from 43 species, constructed using the Neighbor-Joining (NJ) method in MEGA7 based on evolutionary distances. Branch lengths were shown in the same units as the evolutionary distances used for tree construction. The outermost annotation indicated the residue type at the position equivalent to duck C195 in each species. (**B**) Multiple sequence alignment of STING from duck, human, and bovine. Residue numbers above the alignment corresponded to duck STING. Green triangle mark the reference residues at the α1-helix tips used to quantify dimer closure by the Cα–Cα distance: His185 (human), Phe186 (bovine), and Ala188 (duck). Purple triangle mark the reference residues corresponded to duck residues 318–324 (EELVEAE), which form a short α-helix in the duck LBD structure, whereas the aligned segments in bovine/human (315–321; QEPAEGS/QEPADDS) were predominantly coil. The yellow triangles indicate bovine-specific cysteine residues located within the transmembrane domain (TMD), namely C71, C90, C95, C103, and C107. Identical and similar residues were highlighted with red and white boxes, respectively. The alignment was generated using the ESPript 3.0 web server. (**C**) Crystal structure of the human STING LBD in complex with 2′3′-cGAMP (PDB: 4KSY). The symmetric STING dimer was shown as gray cartoon representation, and 2′3′-cGAMP was shown as stick representation, bound within the inter-protomer cleft at the dimer interface. The black dashed box highlighted the region of residues 315–321. (**D**) B-factor–colored representation of 2′3′-cGAMP–bound human STING LBD (PDB: 4KSY), with high B-factors in red (thick ribbons) and low B-factors in green (thin ribbons). The black dashed box highlighted the region of residues 315–321. The ligand 2′3′-cGAMP was displayed as spheres representation.
(DOCX)

S2 Fig The 2Fo–Fc electron-density maps for diABZI3. (**A**) Chemical structure of diABZI3. (**B**-**D**) The 2Fo–Fc electron-density maps for diABZI3, contoured at 1.0 σ after refinement of the duck STING LBD–diABZI3 (**B**), bovine STING LBD–diABZI3 (**C**), and human STING LBD–diABZI3 (**D**) complexes.
(DOCX)

S3 Fig Comparative analysis of STING oligomerization and multiple sequence alignment of STING LBDs across species. (**A**) Non-reducing SDS-PAGE detection of oligomerization of purified human, bovine, and duck STING LBDs under no-ligand, 2′3′-cGAMP, and diABZI3 conditions. (**B**) Domain organization of human, duck, and bovine STING, including chimeric constructs used in this study. (**C**) Multiple sequence alignment of the STING LBDs from duck, human, and bovine. Residue numbers above the alignment corresponded to duck STING. Green triangle marked duck-specific cysteines (C181, C195, C279, and C308). Identical residues were highlighted with red. The alignment was generated using the ESPript 3.0 web server. (**D**) Gel-filtration chromatography of purified duck C195S STING LBD. (**E**) Non-reducing SDS-PAGE detection of oligomerization of purified duck C195S STING LBD under no-ligand, 2′3′-cGAMP, and diABZI3 conditions. (**F**) The sequence logo of STING residues 190–200 is generated with WebLogo based on the sequence alignment of STING from waterfowls.
(DOCX)

S4 Fig Ordered assemblies in published STING structures. (**A**) Side view of the crystal packing of the human STING LBD in complex with 2’3’-cGAMP (PDB ID: 4KSY). (**B**) Side view of the crystal packing of the human STING (H232) LBD variant in complex with 2’3’-cGAMP (PDB ID: 4LOH). (**C**) Side view of the crystal packing of the human STING (A230) LBD variant in complex with 2’3’-cGAMP (PDB ID: 4F5D). (**D**) Side view of the crystal packing of human STING LBD in complex with c-di-AMP (CDA) (PDB ID: 6CFF). (**E**) Side view of the crystal packing of Mouse STING LBD in complex with 2’3’-cGAMP (PDB ID: 4LOJ). (**F**) Side view of the crystal packing of Anemone STING LBD in complex with 2’3’-cGAMP (PDB ID: 5CFQ). (**G**) Side view of the crystal packing of Bacterial STING LBD in complex with c-di-GMP (CDG) (PDB ID: 7EBD). (**H**) Side view of the active conformation of full-length human STING (PDB: 8IK3). (**I**) Orthogonal side views of the autoinhibited conformation of full-length Chicken apo-STING (PDB: 8IK0). All models were shown in surface representation, with each STING protomer in a distinct color.
(DOCX)

S5 Fig Native gel analysis of STING oligomerization. (**A**) Immunoblot analysis of HEK-293T cell lines stably expressing wild-type (WT) or C195S mutant duck STING. GAPDH was used as a loading control. (**B**) Native PAGE analysis of full-length duck STING oligomerization. HEK-293T cells stably expressing WT or C195S duck STING were left unstimulated or stimulated with 2′3′-cGAMP or diABZI3 for 12 h, followed by lysis and separation under non-denaturing conditions to resolve STING oligomeric species. Corresponding SDS–PAGE immunoblots wereshown below to verify comparable STING expression levels, with GAPDH serving as a loading control.
(DOCX)

S6 Fig Mutational and chimeric analyses reveal CTT-dependent control of STING signaling and LBD-mediated regulation of STING oligomerization. (**A**) Multiple sequence alignment of the STING C-terminal tail (CTT) from duck, human, and bovine. Yellow boxes denoted L364 in duck STING and P361 in human STING. Green boxes highlighted the IRF3-binding *p*L*x*IS motif (*p* represents the hydrophilic residue, *x* represents any residue, and S represents the phosphorylation site). Blue boxes indicated the TBK1-binding PLPLRT/SD consensus motif. (**B**) IFN-β luciferase reporter assays in HEK-293T cells expressing duck WT/ duck WT L364P/duck-hCTT STING under untreated, 2′3′-cGAMP treated, and diABZI3 treated conditions. All data represented three biological replicates, error bars were ± SD and significance is determined by Students t-test; * *P* < 0.05, ** *P* < 0.01, *** *P* < 0.001. (**C**) Non-reducing SDS-PAGE/Western blotting analysis of STING oligomerization in DEFs expressing duck WT and C195S. (**D**) Non-reducing SDS-PAGE/Western blotting analysis of oligomerization in HEK-293T cells expressing human, duck, and bovine STING (WT) as well as duck-hCTT and bovine-hCTT after stimulation with diABZI3. Data were representative of three biological replicates. (**E**) STING oligomerization was analyzed by SDS-PAGE/Western blotting in HEK-293T cells expressing bovine STING wild-type (WT), C90S/C95S, C103S/C107S, C71S, and C107S mutants under non-reducing (−DTT, left) and reducing (+DTT, right) conditions. The data were representative of three independent biological replicates.
(DOCX)

S1 Table NCBI accession numbers of STING sequences from 43 species. (DOCX)

S2 Table Data collection and refinement statistics. (DOCX)

S3 Table Sequences of STING residues 190–200 from seven waterfowl species. (DOCX)

S4 Table Key amino acid residues at the Z-shaped tetramer interface of duck STING across 47 species. (DOCX)

S5 Table Key amino acid residues at the head-to-head interface of chicken STING across 47 species. (DOCX)
