# Supplementary material for: A unique Z-shaped tetramer mediates the autoinhibition of waterfowl STING
Source: PLoS Pathog. 2026 Apr 8;22(4):e1014111. doi: 10.1371/journal.ppat.1014111 (PMC13061200; doi:10.1371/journal.ppat.1014111)
Supplement: S1 Fig — (A) Phylogenetic tree of STING sequences from 43 species, constructed using the Neighbor-Joining (NJ) method in MEGA7 based on evolutionary distances. Branch lengths were shown in the same units as the evolutionary distances used for tree construction. The outermost annotation indicated the residue type at the position equivalent to duck C195 in each species. (B) Multiple sequence alignment of STING from duck, human, and bovine. Residue numbers above the alignment corresponded to duck STING. Green triangle mark the reference residues at the α1-helix tips used to quantify dimer closure by the Cα–Cα distance: His185 (human), Phe186 (bovine), and Ala188 (duck). Purple triangle mark the reference residues corresponded to duck residues 318–324 (EELVEAE), which form a short α-helix in the duck LBD structure, whereas the aligned segments in bovine/human (315–321; QEPAEGS/QEPADDS) were predominantly coil. The yellow triangles indicate bovine-specific cysteine residues located within the transmembrane domain (TMD), namely C71, C90, C95, C103, and C107. Identical and similar residues were highlighted with red and white boxes, respectively. The alignment was generated using the ESPript 3.0 web server. (C) Crystal structure of the human STING LBD in complex with 2′3′-cGAMP (PDB: 4KSY). The symmetric STING dimer was shown as gray cartoon representation, and 2′3′-cGAMP was shown as stick representation, bound within the inter-protomer cleft at the dimer interface. The black dashed box highlighted the region of residues 315–321. (D) B-factor–colored representation of 2′3′-cGAMP–bound human STING LBD (PDB: 4KSY), with high B-factors in red (thick ribbons) and low B-factors in green (thin ribbons). The black dashed box highlighted the region of residues 315–321. The ligand 2′3′-cGAMP was displayed as spheres representation. (DOCX) [file ppat.1014111.s001.docx]

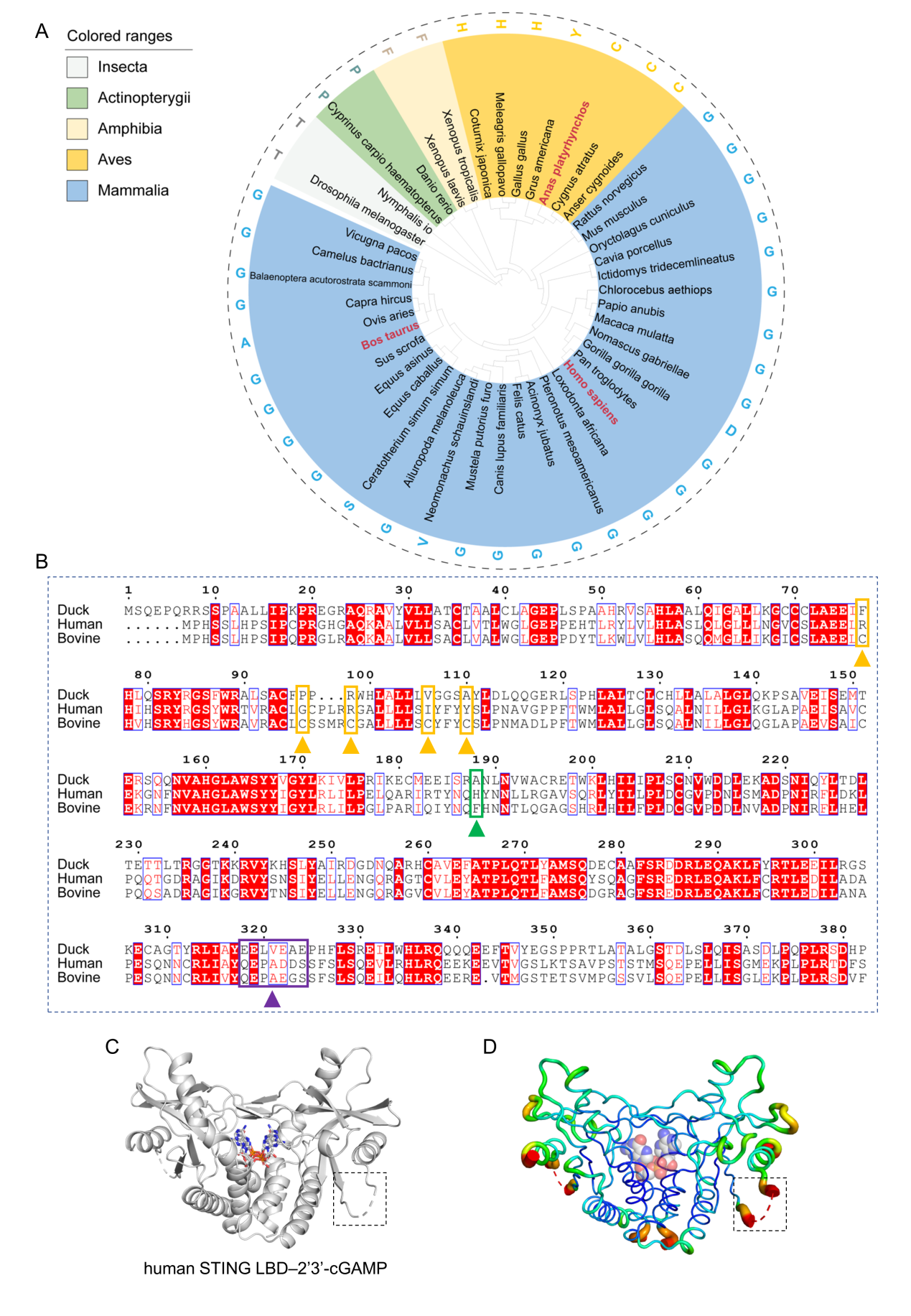


**S1 Fig. Phylogenetic analysis and sequence alignment of STING.**

(**A**) Phylogenetic tree of STING sequences from 43 species, constructed using the Neighbor-Joining (NJ) method in MEGA7 based on evolutionary distances. Branch lengths were shown in the same units as the evolutionary distances used for tree construction. The outermost annotation indicated the residue type at the position equivalent to duck C195 in each species.

(**B**) Multiple sequence alignment of STING from duck, human, and bovine. Residue numbers above the alignment corresponded to duck STING. Green triangle mark the reference residues at the α1-helix tips used to quantify dimer closure by the Cα–Cα distance: His185 (human), Phe186 (bovine), and Ala188 (duck). Purple triangle mark the reference residues corresponded to duck residues 318–324 (EELVEAE), which form a short α-helix in the duck LBD structure, whereas the aligned segments in bovine/human (315–321; QEPAEGS/QEPADDS) were predominantly coil. The yellow triangles indicate bovine-specific cysteine residues located within the transmembrane domain (TMD), namely C71, C90, C95, C103, and C107. Identical and similar residues were highlighted with red and white boxes, respectively. The alignment was generated using the ESPript 3.0 web server.

(**C**) Crystal structure of the human STING LBD in complex with 2′3′-cGAMP (PDB: 4KSY). The symmetric STING dimer was shown as gray cartoon representation, and 2′3′-cGAMP was shown as stick representation, bound within the inter-protomer cleft at the dimer interface. The black dashed box highlighted the region of residues 315–321.

(**D**) B-factor–colored representation of 2′3′-cGAMP–bound human STING LBD (PDB: 4KSY), with high B-factors in red (thick ribbons) and low B-factors in green (thin ribbons). The black dashed box highlighted the region of residues 315–321. The ligand 2′3′-cGAMP was displayed as spheres representation.
