# Supplementary material for: A unique Z-shaped tetramer mediates the autoinhibition of waterfowl STING
Source: PLoS Pathog. 2026 Apr 8;22(4):e1014111. doi: 10.1371/journal.ppat.1014111 (PMC13061200; doi:10.1371/journal.ppat.1014111)
Supplement: S2 Fig — (A) Chemical structure of diABZI3. (B-D) The 2Fo–Fc electron-density maps for diABZI3, contoured at 1.0 σ after refinement of the duck STING LBD–diABZI3 (B), bovine STING LBD–diABZI3 (C), and human STING LBD–diABZI3 (D) complexes. (DOCX) [file ppat.1014111.s002.docx]

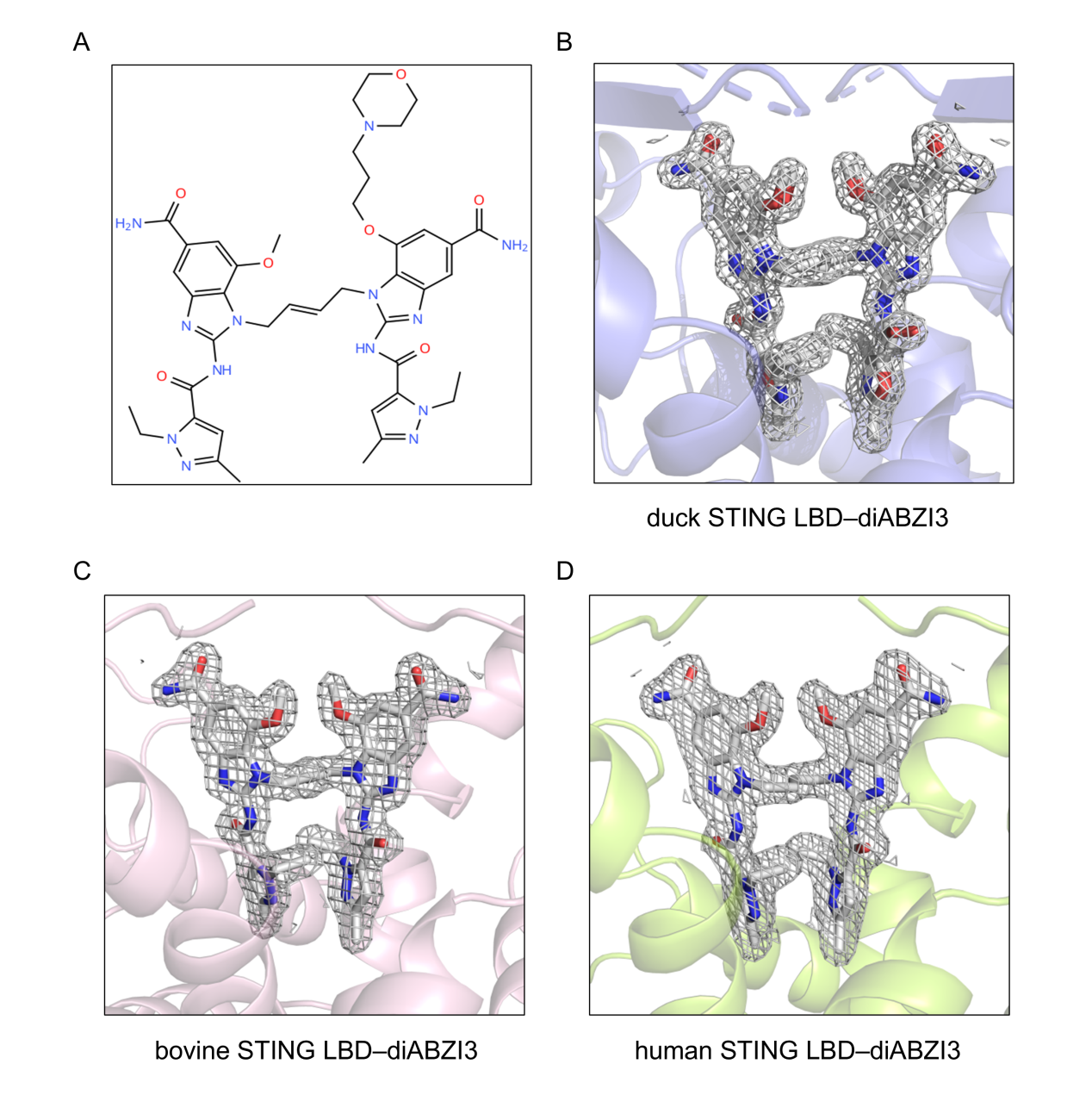


**S2 Fig. The 2Fo–Fc electron-density maps for diABZI3.**

(**A**) Chemical structure of diABZI3.

(**B**-**D**) The 2Fo–Fc electron-density maps for diABZI3, contoured at 1.0 σ after refinement of the duck STING LBD–diABZI3 (**B**), bovine STING LBD–diABZI3 (**C**), and human STING LBD–diABZI3 (**D**) complexes.
