# Supplementary material for: A unique Z-shaped tetramer mediates the autoinhibition of waterfowl STING
Source: PLoS Pathog. 2026 Apr 8;22(4):e1014111. doi: 10.1371/journal.ppat.1014111 (PMC13061200; doi:10.1371/journal.ppat.1014111)
Supplement: S3 Fig — (A) Non-reducing SDS-PAGE detection of oligomerization of purified human, bovine, and duck STING LBDs under no-ligand, 2′3′-cGAMP, and diABZI3 conditions. (B) Domain organization of human, duck, and bovine STING, including chimeric constructs used in this study. (C) Multiple sequence alignment of the STING LBDs from duck, human, and bovine. Residue numbers above the alignment corresponded to duck STING. Green triangle marked duck-specific cysteines (C181, C195, C279, and C308). Identical residues were highlighted with red. The alignment was generated using the ESPript 3.0 web server. (D) Gel-filtration chromatography of purified duck C195S STING LBD. (E) Non-reducing SDS-PAGE detection of oligomerization of purified duck C195S STING LBD under no-ligand, 2′3′-cGAMP, and diABZI3 conditions. (F) The sequence logo of STING residues 190–200 is generated with WebLogo based on the sequence alignment of STING from waterfowls. (DOCX) [file ppat.1014111.s003.docx]

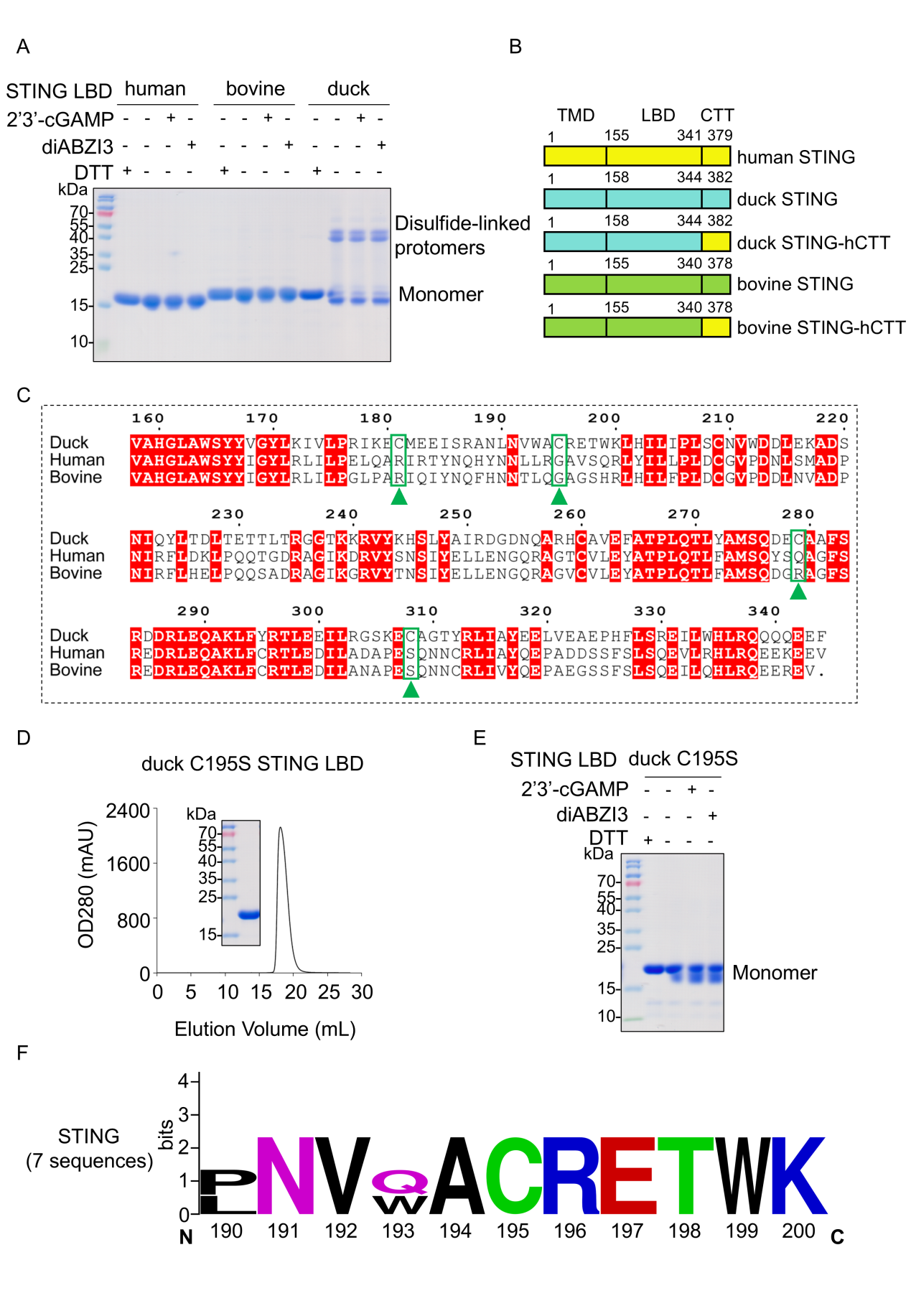


**S3 Fig. Comparative analysis of STING oligomerization and multiple sequence alignment of STING LBDs across species.**

(**A**) Non-reducing SDS-PAGE detection of oligomerization of purified human, bovine, and duck STING LBDs under no-ligand, 2′3′-cGAMP, and diABZI3 conditions.

(**B**) Domain organization of human, duck, and bovine STING, including chimeric constructs used in this study.

(**C**) Multiple sequence alignment of the STING LBDs from duck, human, and bovine. Residue numbers above the alignment corresponded to duck STING. Green triangle marked duck-specific cysteines (C181, C195, C279, and C308). Identical residues were highlighted with red. The alignment was generated using the ESPript 3.0 web server.

(**D**) Gel-filtration chromatography of purified duck C195S STING LBD.

(**E**) Non-reducing SDS-PAGE detection of oligomerization of purified duck C195S STING LBD under no-ligand, 2′3′-cGAMP, and diABZI3 conditions.

(**F**) The sequence logo of STING residues 190–200 is generated with WebLogo based on the sequence alignment of STING from waterfowls.
