# Supplementary material for: A unique Z-shaped tetramer mediates the autoinhibition of waterfowl STING
Source: PLoS Pathog. 2026 Apr 8;22(4):e1014111. doi: 10.1371/journal.ppat.1014111 (PMC13061200; doi:10.1371/journal.ppat.1014111)
Supplement: S4 Fig — (A) Side view of the crystal packing of the human STING LBD in complex with 2’3’-cGAMP (PDB ID: 4KSY). (B) Side view of the crystal packing of the human STING (H232) LBD variant in complex with 2’3’-cGAMP (PDB ID: 4LOH). (C) Side view of the crystal packing of the human STING (A230) LBD variant in complex with 2’3’-cGAMP (PDB ID: 4F5D). (D) Side view of the crystal packing of human STING LBD in complex with c-di-AMP (CDA) (PDB ID: 6CFF). (E) Side view of the crystal packing of Mouse STING LBD in complex with 2’3’-cGAMP (PDB ID: 4LOJ). (F) Side view of the crystal packing of Anemone STING LBD in complex with 2’3’-cGAMP (PDB ID: 5CFQ). (G) Side view of the crystal packing of Bacterial STING LBD in complex with c-di-GMP (CDG) (PDB ID: 7EBD). (H) Side view of the active conformation of full-length human STING (PDB: 8IK3). (I) Orthogonal side views of the autoinhibited conformation of full-length Chicken apo-STING (PDB: 8IK0). All models were shown in surface representation, with each STING protomer in a distinct color. (DOCX) [file ppat.1014111.s004.docx]

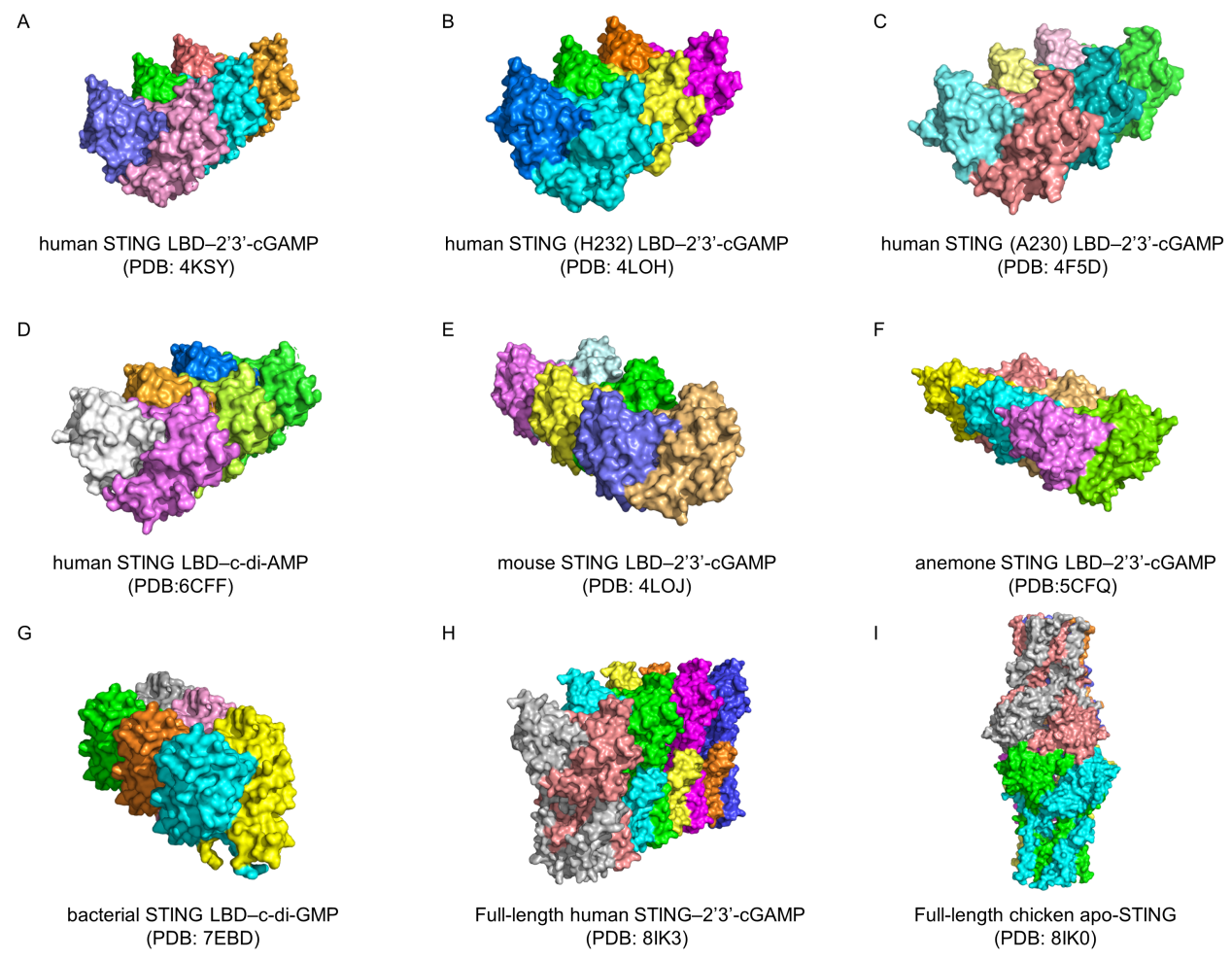


**S4 Fig. Ordered assemblies in published STING structures.**

(**A**) Side view of the crystal packing of the human STING LBD in complex with 2’3’-cGAMP (PDB ID: 4KSY).

(**B**) Side view of the crystal packing of the human STING (H232) LBD variant in complex with 2’3’-cGAMP (PDB ID: 4LOH).

(**C**) Side view of the crystal packing of the human STING (A230) LBD variant in complex with 2’3’-cGAMP (PDB ID: 4F5D).

(**D**) Side view of the crystal packing of human STING LBD in complex with c-di-AMP (CDA) (PDB ID: 6CFF).

(**E**) Side view of the crystal packing of Mouse STING LBD in complex with 2’3’-cGAMP (PDB ID: 4LOJ).

(**F**) Side view of the crystal packing of Anemone STING LBD in complex with 2’3’-cGAMP (PDB ID: 5CFQ).

(**G**) Side view of the crystal packing of Bacterial STING LBD in complex with c-di-GMP (CDG) (PDB ID: 7EBD).

(**H**) Side view of the active conformation of full-length human STING (PDB: 8IK3).

(**I**) Orthogonal side views of the autoinhibited conformation of full-length Chicken apo-STING (PDB: 8IK0). All models were shown in surface representation, with each STING protomer in a distinct color.
