# Supplementary material for: A unique Z-shaped tetramer mediates the autoinhibition of waterfowl STING
Source: PLoS Pathog. 2026 Apr 8;22(4):e1014111. doi: 10.1371/journal.ppat.1014111 (PMC13061200; doi:10.1371/journal.ppat.1014111)
Supplement: S5 Fig — (A) Immunoblot analysis of HEK-293T cell lines stably expressing wild-type (WT) or C195S mutant duck STING. GAPDH was used as a loading control. (B) Native PAGE analysis of full-length duck STING oligomerization. HEK-293T cells stably expressing WT or C195S duck STING were left unstimulated or stimulated with 2′3′-cGAMP or diABZI3 for 12 h, followed by lysis and separation under non-denaturing conditions to resolve STING oligomeric species. Corresponding SDS–PAGE immunoblots wereshown below to verify comparable STING expression levels, with GAPDH serving as a loading control. (DOCX) [file ppat.1014111.s005.docx]

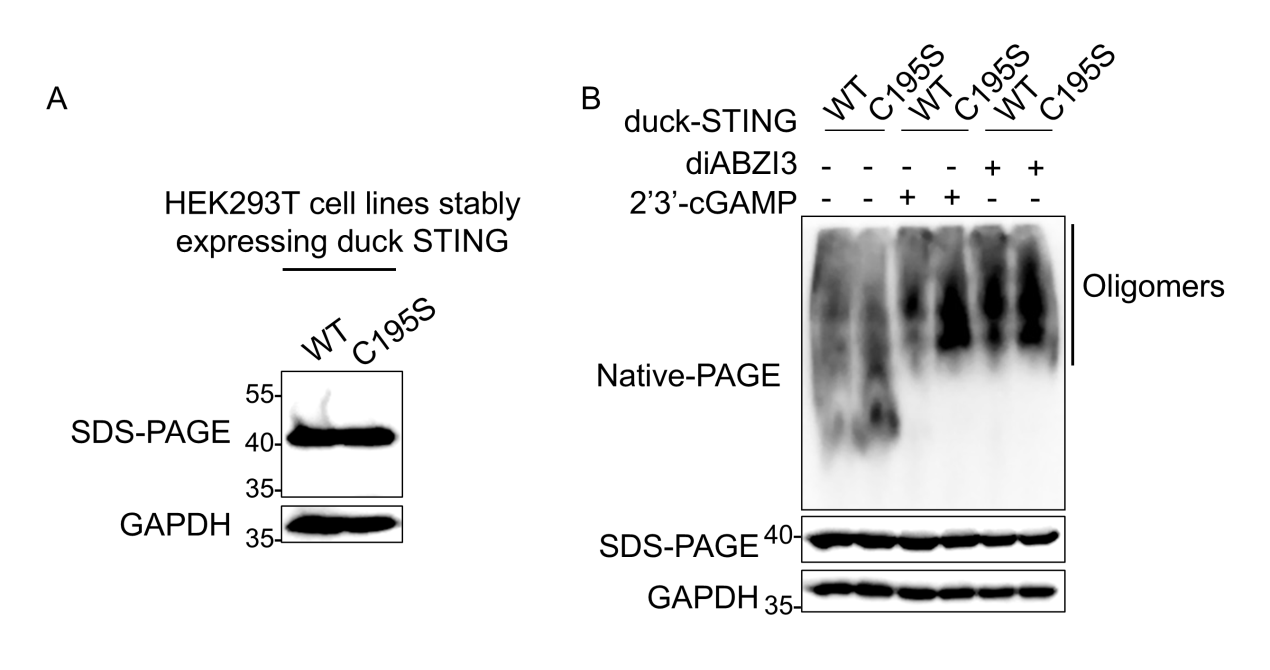


**S5 Fig. Native gel analysis of STING oligomerization**.

(**A**) Immunoblot analysis of HEK-293T cell lines stably expressing wild-type (WT) or C195S mutant duck STING. GAPDH was used as a loading control.

(**B**) Native PAGE analysis of full-length duck STING oligomerization. HEK-293T cells stably expressing WT or C195S duck STING were left unstimulated or stimulated with 2′3′-cGAMP or diABZI3 for 12 h, followed by lysis and separation under non-denaturing conditions to resolve STING oligomeric species. Corresponding SDS–PAGE immunoblots wereshown below to verify comparable STING expression levels, with GAPDH serving as a loading control.
