# Supplementary material for: A unique Z-shaped tetramer mediates the autoinhibition of waterfowl STING
Source: PLoS Pathog. 2026 Apr 8;22(4):e1014111. doi: 10.1371/journal.ppat.1014111 (PMC13061200; doi:10.1371/journal.ppat.1014111)
Supplement: S6 Fig — (A) Multiple sequence alignment of the STING C-terminal tail (CTT) from duck, human, and bovine. Yellow boxes denoted L364 in duck STING and P361 in human STING. Green boxes highlighted the IRF3-binding pLxIS motif (p represents the hydrophilic residue, x represents any residue, and S represents the phosphorylation site). Blue boxes indicated the TBK1-binding PLPLRT/SD consensus motif. (B) IFN-β luciferase reporter assays in HEK-293T cells expressing duck WT/ duck WT L364P/duck-hCTT STING under untreated, 2′3′-cGAMP treated, and diABZI3 treated conditions. All data represented three biological replicates, error bars were ± SD and significance is determined by Students t-test; * P < 0.05, ** P < 0.01, *** P < 0.001. (C) Non-reducing SDS-PAGE/Western blotting analysis of STING oligomerization in DEFs expressing duck WT and C195S. (D) Non-reducing SDS-PAGE/Western blotting analysis of oligomerization in HEK-293T cells expressing human, duck, and bovine STING (WT) as well as duck-hCTT and bovine-hCTT after stimulation with diABZI3. Data were representative of three biological replicates. (E) STING oligomerization was analyzed by SDS-PAGE/Western blotting in HEK-293T cells expressing bovine STING wild-type (WT), C90S/C95S, C103S/C107S, C71S, and C107S mutants under non-reducing (−DTT, left) and reducing (+DTT, right) conditions. The data were representative of three independent biological replicates. (DOCX) [file ppat.1014111.s006.docx]

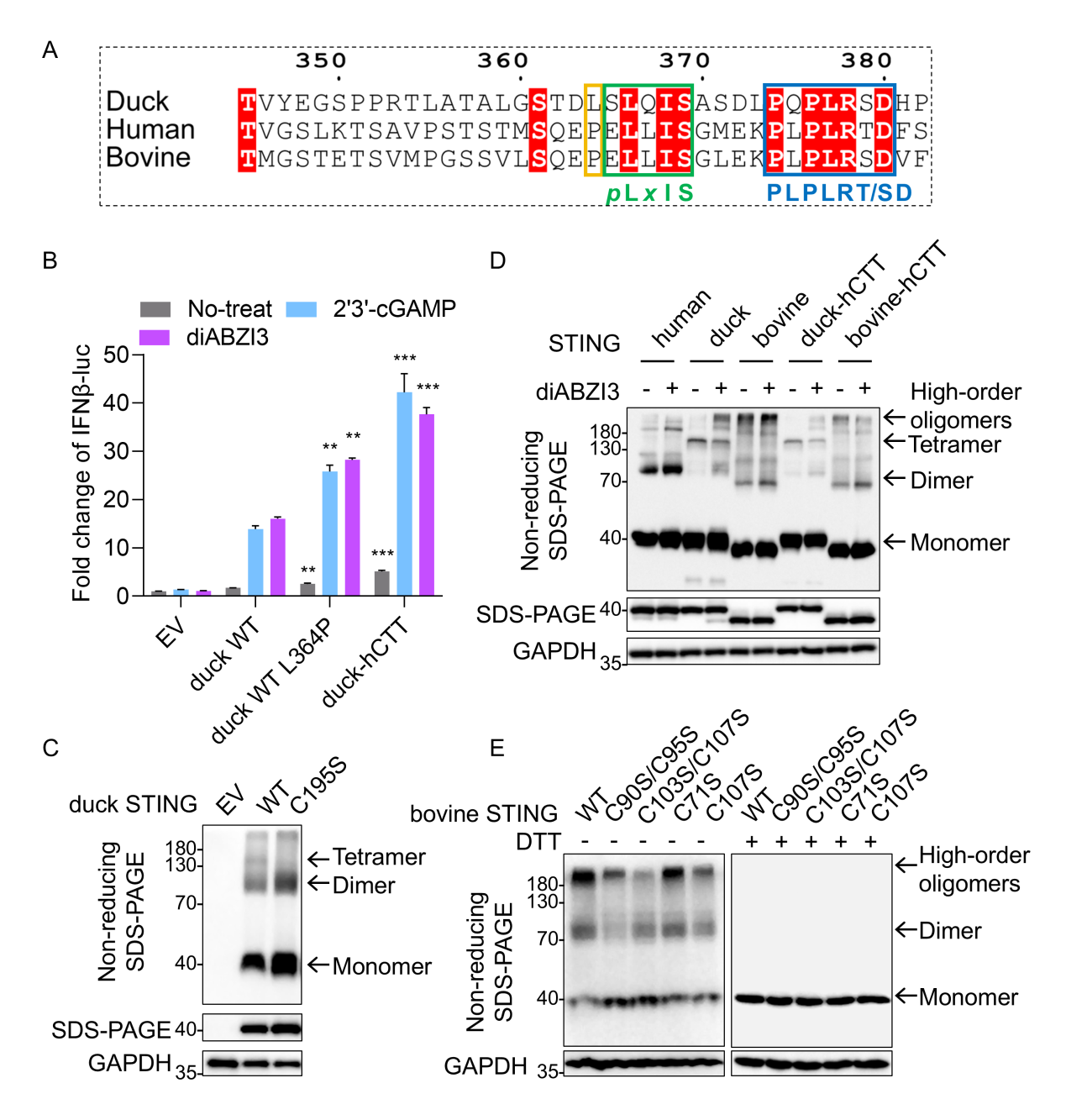


**S6 Fig. Mutational and chimeric analyses reveal CTT-dependent control of STING signaling and LBD-mediated regulation of STING oligomerization.**

(**A**) Multiple sequence alignment of the STING C-terminal tail (CTT) from duck, human, and bovine. Yellow boxes denoted L364 in duck STING and P361 in human STING. Green boxes highlighted the IRF3-binding pLxIS motif (p represents the hydrophilic residue, x represents any residue, and S represents the phosphorylation site). Blue boxes indicated the TBK1-binding PLPLRT/SD consensus motif.

(**B**) IFN-β luciferase reporter assays in HEK-293T cells expressing duck WT/ duck WT L364P/duck-hCTT STING under untreated, 2′3′-cGAMP treated, and diABZI3 treated conditions. All data represented three biological replicates, error bars were ± SD and significance is determined by Students t-test; **P*<0.05, ** *P*<0.01, *** *P*<0.001.

(**C**) Non-reducing SDS-PAGE/Western blotting analysis of STING oligomerization in DEFs expressing duck WT and C195S.

(**D**) Non-reducing SDS-PAGE/Western blotting analysis of oligomerization in HEK-293T cells expressing human, duck, and bovine STING (WT) as well as duck-hCTT and bovine-hCTT after stimulation with diABZI3. Data were representative of three biological replicates.

(**E**) STING oligomerization was analyzed by SDS-PAGE/Western blotting in HEK-293T cells expressing bovine STING wild-type (WT), C90S/C95S, C103S/C107S, C71S, and C107S mutants under non-reducing (−DTT, left) and reducing (+DTT, right) conditions. The data were representative of three independent biological replicates.
