# Supplementary material for: A unique Z-shaped tetramer mediates the autoinhibition of waterfowl STING
Source: PLoS Pathog. 2026 Apr 8;22(4):e1014111. doi: 10.1371/journal.ppat.1014111 (PMC13061200; doi:10.1371/journal.ppat.1014111)
Supplement: S1 Table — (DOCX) [file ppat.1014111.s007.docx]

**S1 Table. NCBI accession numbers of STING sequences from 43 species.**

| **Species** | **NCBI Entry** |
| --- | --- |
| *Acinonyx jubatus* | XP_014919539.1 |
| *Ailuropoda melanoleuca* | XP_002912620.1 |
| *Anas platyrhynchos* | XP_027323921.2 |
| *Anser cygnoides* | XP_013057485.3 |
| *Balaenoptera acutorostrata scammoni* | XP_007172318.1 |
| *Bos taurus* | ANQ45218.1 |
| *Camelus bactrianus* | NP_001306707.1 |
| *Canis lupus familiaris* | XP_005617314.1 |
| *Capra hircus* | NP_001306207.1 |
| *Cavia porcellus* | XP_003477199.1 |
| *Ceratotherium simum simum* | XP_014650944.1 |
| *Chlorocebus aethiops* | UQV25891.1 |
| *Coturnix japonica* | XP_015731739.1 |
| *Cygnus atratus* | XP_035415548.1 |
| *Cyprinus carpio haematopterus* | WKE35538.1 |
| *Danio rerio* | XP_005157178.1 |
| *Drosophila melanogaster* | NP_610525.4 |
| *Equus asinus* | XP_014709351.1 |
| *Equus caballus* | XP_005599422.1 |
| *Felis catus* | XP_023111467.1 |
| *Gallus gallus* | XP_046783054.1 |
| *Gorilla gorilla gorilla* | XP_004042660.2 |
| *Grus americana* | NWH21501.1 |
| *Homo sapiens* | AVQ94753.1 |
| *Ictidomys tridecemlineatus* | XP_005327332.1 |
| *Loxodonta africana* | XP_003404845.1 |
| *Macaca mulatta* | XP_014996496.2 |
| *Meleagris gallopavo* | XP_010717095.1 |
| *Mus musculus* | NP_082537.1 |
| *Mustela putorius furo* | XP_012907883.1 |
| *Neomonachus schauinslandi* | XP_021557627.1 |
| *Nomascus gabriellae* | AVQ94740.1 |
| *Nymphalis io* | XP_050347592.1 |
| *Oryctolagus cuniculus* | XP_002710295.1 |
| *Ovis aries* | XP_004008906.1 |
| *Pan troglodytes* | XP_001135484.1 |
| *Papio anubis* | XP_003900232.1 |
| *Pteronotus mesoamericanus* | XP_054423939.1 |
| *Rattus norvegicus* | NP_001102592.1 |
| *Sus scrofa* | NP_001136310.1 |
| *Vicugna pacos* | XP_015094987.1 |
| *Xenopus laevis* | XP_018110460.1 |
| *Xenopus tropicalis* | NP_001106445.2 |
