# Supplementary material for: A unique Z-shaped tetramer mediates the autoinhibition of waterfowl STING
Source: PLoS Pathog. 2026 Apr 8;22(4):e1014111. doi: 10.1371/journal.ppat.1014111 (PMC13061200; doi:10.1371/journal.ppat.1014111)
Supplement: S2 Table — (DOCX) [file ppat.1014111.s008.docx]

**S2 Table. Data collection and refinement statistics.**

|  | duck－2′3′-cGAMP | bovine－2′3′-cGAMP | duck－diABZI3 | bovine－diABZI3 | human－diABZI3 |
| --- | --- | --- | --- | --- | --- |
| **Protein Data Bank ID** | 9WH7 | 9WHA | 9WHF | 9WH9 | 9WH8 |
| **Data collection** |  |  |  |  |  |
| Wavelength (Å) | 0.9792 | 0.9792 | 0.9791 | 0.9792 | 0.9792 |
| Space group | P6₅22 | P2₁2₁2₁ | P4₁2₁2 | P2₁2₁2₁ | P6₁22 |
| Cell dimensions |  |  |  |  |  |
| *a, b, c* (Å) | 83.535, 83.535, 353.159 | 50.729, 57.910, 132.269 | 67.050, 67.050, 98.070 | 44.997, 57.544, 136.378 | 62.763, 62.763, 219.376 |
| *α, β, γ* | 90.00, 90.00 120.00 | 90.00, 90.00, 90.00 | 90.00 90.00 90.00 | 90.00, 90.00, 90.00 | 90.00, 90.00, 120.00 |
| Resolution (Å) | 88.29-2.49  (2.60-2.49) | 66.13-1.60  (1.63-1.60) | 67.05-1.65  (1.68-1.65) | 68.19-1.81  (1.91-1.81) | 219.38-1.90  (1.95-1.90) |
| *R_merge_* (%) | 21.5 (208.8) | 19.3 (108.8) | 13.3 (287.4) | 10.2 (208.8) | 8.7 (139.0) |
| *I*/*σI* | 13.5 (2.5) | 7.5 (1.5) | 17.1 (1.5) | 11.9 (1.0) | 21.5 (3.4) |
| Completeness (%) | 99.7 (97.7) | 99.8 (99.8) | 100.0 (100.0) | 98.7 (91.2) | 100.0 (100.0) |
| Redundancy | 30.1 (27.2) | 11.2 (11.0) | 25.6 (26.6) | 11.4 (7.4) | 36.6 (36.7) |
| **Refinement** |  |  |  |  |  |
| No. of reflections | 26426 | 51886 | 27609 | 32251 | 21138 |
| *R_work_*/*R_free_* (%) | 0.194/0.231 | 0.216/0.241 | 0.194/0.215 | 0.200/0.258 | 0.210/0.263 |
| No. of atoms |  |  |  |  |  |
| Protein | 3076 | 2972 | 1490 | 2854 | 1387 |
| Ligand | 90 | 90 | 54 | 54 | 54 |
| Water | 52 | 284 | 188 | 124 | 94 |
| B-factors (Å^2^) |  |  |  |  |  |
| Protein | 63.4 | 33.9 | 38.7 | 50.7 | 55.3 |
| Ligand | 46.2 | 15.4 | 18.0 | 26.9 | 29.1 |
| Water | 53.4 | 38.8 | 43.8 | 46.7 | 54.0 |
| **Root mean square deviation** |  |  |  |  |  |
| Bonds lengths (Å) | 0.002 | 0.003 | 0.004 | 0.006 | 0.007 |
| Bonds angles (°) | 0.525 | 0.687 | 0.757 | 0.995 | 0.941 |

Values in parenthesis corresponded to the highest-resolution shell
