# Supplementary material for: A unique Z-shaped tetramer mediates the autoinhibition of waterfowl STING
Source: PLoS Pathog. 2026 Apr 8;22(4):e1014111. doi: 10.1371/journal.ppat.1014111 (PMC13061200; doi:10.1371/journal.ppat.1014111)
Supplement: S3 Table — (DOCX) [file ppat.1014111.s009.docx]

**S3 Table. Sequences of STING residues 190–200 from seven waterfowl species.**

| **Species** | **NCBI Entry** | **Sequence** |
| --- | --- | --- |
| *Anas acuta* | XP_068554831.1 | LNVWACRETWK |
| *Anas platyrhynchos* | XP_027323921.2 | LNVWACRETWK |
| *Anser cygnoides* | XP_013057485.3 | PNVQACRETWK |
| *Aythya fuligula* | XP_032053136.1 | LNVWACRETWK |
| *Cygnus atratus* | XP_035415548.1 | PNVQACRETWK |
| *Cygnus olor* | XP_040429588.1 | PNVQACRETWK |
| *Oxyura jamaicensis* | XP_035194429.1 | PNVQACRETWK |

C195 was highlighted in red.
