# Supplementary material for: A unique Z-shaped tetramer mediates the autoinhibition of waterfowl STING
Source: PLoS Pathog. 2026 Apr 8;22(4):e1014111. doi: 10.1371/journal.ppat.1014111 (PMC13061200; doi:10.1371/journal.ppat.1014111)
Supplement: S4 Table — (DOCX) [file ppat.1014111.s010.docx]

**S4 Table. Key amino acid residues at the Z-shaped tetramer interface of duck STING across 47 species.**

| **Class** | **Species** | | **Position (duck STING numbering)** | | | | | | | |
| --- | --- | --- | --- | --- | --- | --- | --- | --- | --- | --- |
|  |  |  | **187** | **191** | **195** | **196** | **251** | **254** | **337** | **342** |
| Aves | Waterfowl | *Anser cygnoides* | K | N | C | R | R | D | R | E |
|  |  | *Cygnus atratus* | K | N | C | R | R | D | R | E |
|  |  | *Anas platyrhynchos* | R | N | C | R | R | D | R | E |
|  |  | *Anas acuta* | R | N | C | R | R | D | R | E |
|  |  | *Aythya fuligula* | R | N | C | R | R | D | R | E |
|  |  | *Cygnus olor* | K | N | C | R | R | D | R | E |
|  |  | *Oxyura jamaicensis* | R | N | C | R | R | D | R | E |
|  | *Grus americana* | | R | N | Y | R | R | D | Q | E |
|  | *Gallus gallus* | | R | M | H | R | R | D | Q | E |
|  | *Meleagris gallopavo* | | R | M | H | H | R | D | Q | E |
|  | *Coturnix japonica* | | R | I | H | R | R | D | Q | E |
| Mammalia | *Rattus norvegicus* | | Q | N | G | A | L | G | R | E |
|  | *Mus musculus* | | Q | N | G | A | L | G | R | E |
|  | *Oryctolagus cuniculus* | | Q | N | G | M | L | G | R | E |
|  | *Cavia porcellus* | | K | N | G | T | L | G | R | E |
|  | *Ictidomys tridecemlineatus* | | Q | N | G | A | L | G | R | E |
|  | *Chlorocebus aethiops* | | Q | H | G | A | L | G | R | E |
|  | *Papio anubis* | | Q | N | G | A | L | G | R | E |
|  | *Macaca mulatta* | | Q | N | G | A | L | G | R | E |
|  | *Nomascus gabriellae* | | Q | N | G | A | L | G | R | E |
|  | *Gorilla gorilla gorilla* | | Q | N | D | A | L | G | R | E |
|  | *Pan troglodytes* | | Q | N | G | A | L | G | R | E |
|  | *Homo sapiens* | | Q | N | G | A | L | G | R | E |
|  | *Loxodonta africana* | | L | N | G | A | L | G | R | E |
|  | *Pteronotus mesoamericanus* | | Q | N | G | A | L | G | K | E |
|  | *Acinonyx jubatus* | | Q | N | G | T | L | G | R | E |
|  | *Felis catus* | | Q | N | G | T | L | G | R | E |
|  | *Canis lupus familiaris* | | - | N | G | I | L | G | R | E |
|  | *Mustela putorius furo* | | L | N | G | L | L | G | R | E |
|  | *Neomonachus schauinslandi* | | Q | N | V | I | L | G | R | E |
|  | *Ailuropoda melanoleuca* | | R | N | G | V | L | G | R | E |
|  | *Ceratotherium simum simum* | | E | N | S | A | L | G | R | E |
|  | *Equus caballus* | | Q | N | G | A | L | G | R | E |
|  | *Equus asinus* | | Q | N | G | A | L | G | R | E |
|  | *Sus scrofa* | | Q | N | G | I | L | G | R | E |
|  | *Bos taurus* | | Q | N | G | A | L | G | R | E |
|  | *Ovis aries* | | Q | N | A | A | L | G | R | E |
|  | *Capra hircus* | | Q | N | G | A | L | G | R | E |
|  | *Balaenoptera acutorostrata scammoni* | | Q | N | G | A | L | G | R | E |
|  | *Camelus bactrianus* | | R | N | G | P | L | G | R | E |
|  | *Vicugna pacos* | | R | N | G | P | L | G | R | E |
| **Class** | **Species** | | **Position (duck STING numbering)** | | | | | | | |
|  |  |  | **187** | **191** | **195** | **196** | **251** | **254** | **337** | **342** |
| Insecta | *Drosophila melanogaster* | | M | K | T | F | K | N | Q | - |
|  | *Nymphalis io* | | I | K | I | F | Y | G | R | - |
| Actinopterygii | *Cyprinus carpio haematopterus* | | S | K | P | - | T | - | K | E |
|  | *Danio rerio* | | R | R | P | - | T | - | K | E |
| Amphibia | *Xenopus laevis* | | E | N | F | P | L | D | R | E |
|  | *Xenopus tropicalis* | | K | N | F | P | L | D | K | E |
