# Supplementary material for: A unique Z-shaped tetramer mediates the autoinhibition of waterfowl STING
Source: PLoS Pathog. 2026 Apr 8;22(4):e1014111. doi: 10.1371/journal.ppat.1014111 (PMC13061200; doi:10.1371/journal.ppat.1014111)
Supplement: S5 Table — (DOCX) [file ppat.1014111.s011.docx]

**S5 Table. Key amino acid residues at the head-to-head interface of chicken STING across 47 species.**

| **Class** | **Species** | | **Position (chicken STING numbering)** | | | | | | |
| --- | --- | --- | --- | --- | --- | --- | --- | --- | --- |
|  |  |  | **230** | **231** | **187** | **193** | **194** | **227** | **228** |
| Aves | Waterfowl | *Anser cygnoides* | L | T | I | N | V | L | T |
|  |  | *Cygnus atratus* | L | T | I | N | V | L | T |
|  |  | *Anas platyrhynchos* | L | T | I | N | V | L | T |
|  |  | *Anas acuta* | L | T | I | N | V | L | T |
|  |  | *Aythya fuligula* | L | T | I | N | V | L | T |
|  |  | *Cygnus olor* | L | T | I | N | V | L | T |
|  |  | *Oxyura jamaicensis* | L | T | I | N | V | L | T |
|  | *Grus americana* | | L | T | F | N | L | L | T |
|  | *Gallus gallus* | | L | P | I | M | L | L | A |
|  | *Meleagris gallopavo* | | L | P | I | M | L | L | A |
|  | *Coturnix japonica* | | L | P | I | I | L | L | A |
| Mammalia | *Rattus norvegicus* | | L | P | F | N | M | R | D |
|  | *Mus musculus* | | L | P | F | N | M | R | D |
|  | *Oryctolagus cuniculus* | | L | P | Y | N | I | L | H |
|  | *Cavia porcellus* | | L | P | Y | N | T | L | H |
|  | *Ictidomys tridecemlineatus* | | L | P | Y | N | M | L | H |
|  | *Chlorocebus aethiops* | | L | P | Y | H | L | L | D |
|  | *Papio anubis* | | L | P | Y | N | L | L | D |
|  | *Macaca mulatta* | | L | P | Y | N | L | L | D |
|  | *Nomascus gabriellae* | | L | P | Y | N | L | L | D |
|  | *Gorilla gorilla gorilla* | | L | P | Y | N | L | L | D |
|  | *Pan troglodytes* | | L | P | Y | N | L | L | D |
|  | *Homo sapiens* | | L | P | Y | N | L | L | D |
|  | *Loxodonta africana* | | L | P | Y | N | M | V | Q |
|  | *Pteronotus mesoamericanus* | | L | P | Y | N | L | L | H |
|  | *Acinonyx jubatus* | | L | P | C | N | I | L | Y |
|  | *Felis catus* | | L | P | C | N | I | L | Y |
|  | *Canis lupus familiaris* | | L | P | L | N | M | L | Y |
|  | *Mustela putorius furo* | | L | P | Y | N | M | L | Y |
|  | *Neomonachus schauinslandi* | | L | P | Y | N | M | L | Y |
|  | *Ailuropoda melanoleuca* | | L | P | Y | N | M | L | Y |
|  | *Ceratotherium simum simum* | | L | P | Y | N | V | L | H |
|  | *Equus caballus* | | L | P | Y | N | V | L | H |
|  | *Equus asinus* | | L | P | Y | N | V | L | H |
|  | *Sus scrofa* | | L | P | Y | N | V | L | H |
|  | *Bos taurus* | | L | P | Y | N | T | L | H |
|  | *Ovis aries* | | L | P | Y | N | T | L | H |
|  | *Capra hircus* | | L | P | Y | N | T | L | H |
|  | *Balaenoptera acutorostrata scammoni* | | L | P | Y | N | V | L | H |
|  | *Camelus bactrianus* | | L | P | Y | N | V | L | N |
|  | *Vicugna pacos* | | L | P | Y | N | V | L | N |
| **Class** | **Species** | | **Position (duck STING numbering)** | | | | | | |
|  |  |  | **230** | **231** | **187** | **193** | **194** | **227** | **228** |
| Insecta | *Drosophila melanogaster* | | L | E | L | K | N | A | E |
|  | *Nymphalis io* | | L | E | I | K | Q | L | P |
| Actinopterygii | *Cyprinus carpio haematopterus* | | L | P | Y | K | V | H | E |
|  | *Danio rerio* | | L | P | Y | R | L | H | E |
| Amphibia | *Xenopus laevis* | | L | P | F | N | L | L | K |
|  | *Xenopus tropicalis* | | L | P | F | N | L | L | K |
